# Perturbation of the yeast mitochondrial lipidome and associated membrane proteins following heterologous expression of Artemia-ANT

**DOI:** 10.1038/s41598-018-24305-2

**Published:** 2018-04-12

**Authors:** Emily Chen, Michael A. Kiebish, Justice McDaniel, Katarzyna Niedzwiecka, Roza Kucharczyk, Dora Ravasz, Fei Gao, Niven R. Narain, Rangaprasad Sarangarajan, Thomas N. Seyfried, Vera Adam-Vizi, Christos Chinopoulos

**Affiliations:** 1 BERG LLC, Framingham, MA 01701 USA; 2 Institute of Biochemistry and Biophysics, Polish Academy of Sciences, Warsaw, 02-106 Poland; 3 Department of Medical Biochemistry, Semmelweis University, Budapest, 1094 Hungary; 4 MTA-SE Lendület Neurobiochemistry Research Group, Budapest, 1094 Hungary; 5 Biology Department, Boston College, Chestnut Hill, Boston, MA 02467 USA; 6 MTA-SE Laboratory for Neurobiochemistry, Budapest, 1094 Hungary

## Abstract

Heterologous expression is a landmark technique for studying a protein itself or its effect on the expression host, in which membrane-embedded proteins are a common choice. Yet, the impact of inserting a foreign protein to the lipid environment of host membranes, has never been addressed. Here we demonstrated that heterologous expression of the *Artemia franciscana* adenine nucleotide translocase (ANT) in yeasts altered lipidomic composition of their inner mitochondrial membranes. Along with this, activities of complex II, IV and ATP synthase, all membrane-embedded components, were significantly decreased while their expression levels remained unaffected. Although the results represent an individual case of expressing a crustacean protein in yeast inner mitochondrial membranes, it cannot be excluded that host lipidome alterations is a more widespread epiphenomenon, potentially biasing heterologous expression experiments. Finally, our results raise the possibility that not only lipids modulate protein function, but also membrane-embedded proteins modulate lipid composition, thus revealing a reciprocal mode of regulation for these two biomolecular entities.

## Introduction

Heterologous expression yielding a foreign protein in a host is a widely used molecular biology tool^1,2^. Applications benefiting from this tool are diverse, ranging from studying the protein itself (*i.e*. by patch clamp if it is a channel), identifying its binding partners, or studying the impact of the protein on host viability. Alternatively, the heterologously expressed protein may be fluorescent and responsive to a particular variable (*i.e*. intracellular Ca^2+^ or pH) and/or is targeted towards a particular compartment, or it is intentionally overexpressed for the purpose of protein purification or it is plasma membrane-targeted for raising ligands or antibodies against it. The latter application is inherently relevant to the pharmaceutical industry, because membrane proteins are the targets of >50% of drugs despite representing only ~1% of total cellular proteins^2^. It is therefore not surprising that a Pubmed search for the string [“heterologous expression” OR “exogenous expression” OR “ectopic expression”] yields >24,000 hits. However, among these thousands of studies and despite the fact that many have addressed the lipid composition of yeasts and the organelles within^3–7^ the impact of ‘forcing’ large amounts of a protein on the lipids of the embedding membrane has been addressed from only a specific point of view: candidate genes that are –directly or indirectly- involved in lipid metabolism have been largely identified by heterologous expression in yeasts, followed by tracing the changes in host lipidome^8^. Furthermore, and in a more indirect way, the effects of various growth conditions that are bound to yield changes in protein expression have been queried on the yeast lipidome^3^. Alas, the impact of heterologously expressing a functional, membrane-embedded protein on the host membrane lipidome has been rather oversighted.

By definition, lipids govern the folding, organization, and final structure of all membranes, and they do so by regulating the function of membrane-embedded proteins and those interacting with other membranous surfaces^9–12^. Furthermore, some lipids act as signaling molecules while others participate in the post-translation modification of proteins^11^. The diversification of lipid classes is immense; lipids exhibit an extremely wide spectrum of physicochemical properties stemming from a heterogeneous composition^11^. Lipid composition diversity is reflected from the numerous entries in the ‘LIPID Metabolites And Pathways Strategy’ gateway (LIPID MAPS, http://www.lipidmaps.org/)^13^, currently hosting >40,000 lipid structures.

The function of lipids is to dynamically interact in order to allow for the formation of transient arrangements exhibiting both perpendicular and parallel asymmetry to the plane of the lipid bilayer, thus, they are not covalently bound in membranes. Having said that, it is important to emphasize that some lipids merely bind embedded proteins, while others modulate their structure and/or function^12^. A notable example of lipid modulation on a protein is the requirement for phosphatidylethanolamine (PE) in the folding of the integral membrane protein lactose permease (LacY) of *E. coli*^14^: in the absence of PE, LacY becomes misfolded. Furthermore, the lipid environment determines the orientation of transmembrane domains for several secondary transporters of *E. coli*^15^. Alterations in membrane protein topological rearrangements in response to lipid modulations such as these are not only known to occur^16–18^, but do so rapidly following a threshold change^19^.

However, despite the recognition that membrane lipids influence protein function, the inverse situation, *i.e*. the impact of membrane proteins on lipid composition has never been investigated. Addressing this lack of knowledge becomes even more pressing in light of the discovery of mitochondria-associated membranes (MAMs), specialized membrane subdomains that mediate the exchange of ions and complex lipids between the endoplasmic reticulum and mitochondria^20–22^. Here we heterologously expressed the adenine nucleotide translocase normally found only in the mitochondria of the extremophile invertebrate *Artemia franciscana* (ArANT)^23^, in *Saccharomyces cerevisiae*, in lieu of the endogenous adenine nucleotide translocase AAC2, normally found in yeast. The original aim of that experiment was to identify potential binding partners of the ArANT, a protein expressed in an organism known not to exhibit the phenomenon of permeability transition^24^. Permeability transition of mitochondria is characterized by a Ca^2+^-induced pore opening that allows the flux of metabolites with a molecular weight of up to 1,500 Da across the inner mitochondrial membrane. The ANT is not a structural component of this pore^25^. Recently, ATP synthase has been incriminated as a structural entity of the pore^26–28^ though ANT1 is the voltage sensor^29^, thus, its interaction with genuine pore components is extremely likely. This is especially probable in view of the findings by the group of Houštěk, namely the interaction of the ATP synthase with the ANT^30^. However, the focus of our original experiments shifted when we realized that the heterologous expression of ArANT in yeasts conferred a dramatic alteration in the yeast mitochondrial and mitoplastic lipidome, and this was associated with changes in the functions of other inner mitochondrial membrane-embedded proteins, specifically complexes II, IV and F_o_-F_1_ ATP synthase. At this junction it must be stressed that it is not known if the changes in ETC complex activities were due to the alterations in inner mitochondrial membrane lipidome, or ArANT had some other perturbing effect implying that lipidomic changes weren’t causal.

## Results

### Heterologous expression of ArANT in yeasts perturbs the mitochondrial lipidome

**Figure 1:**
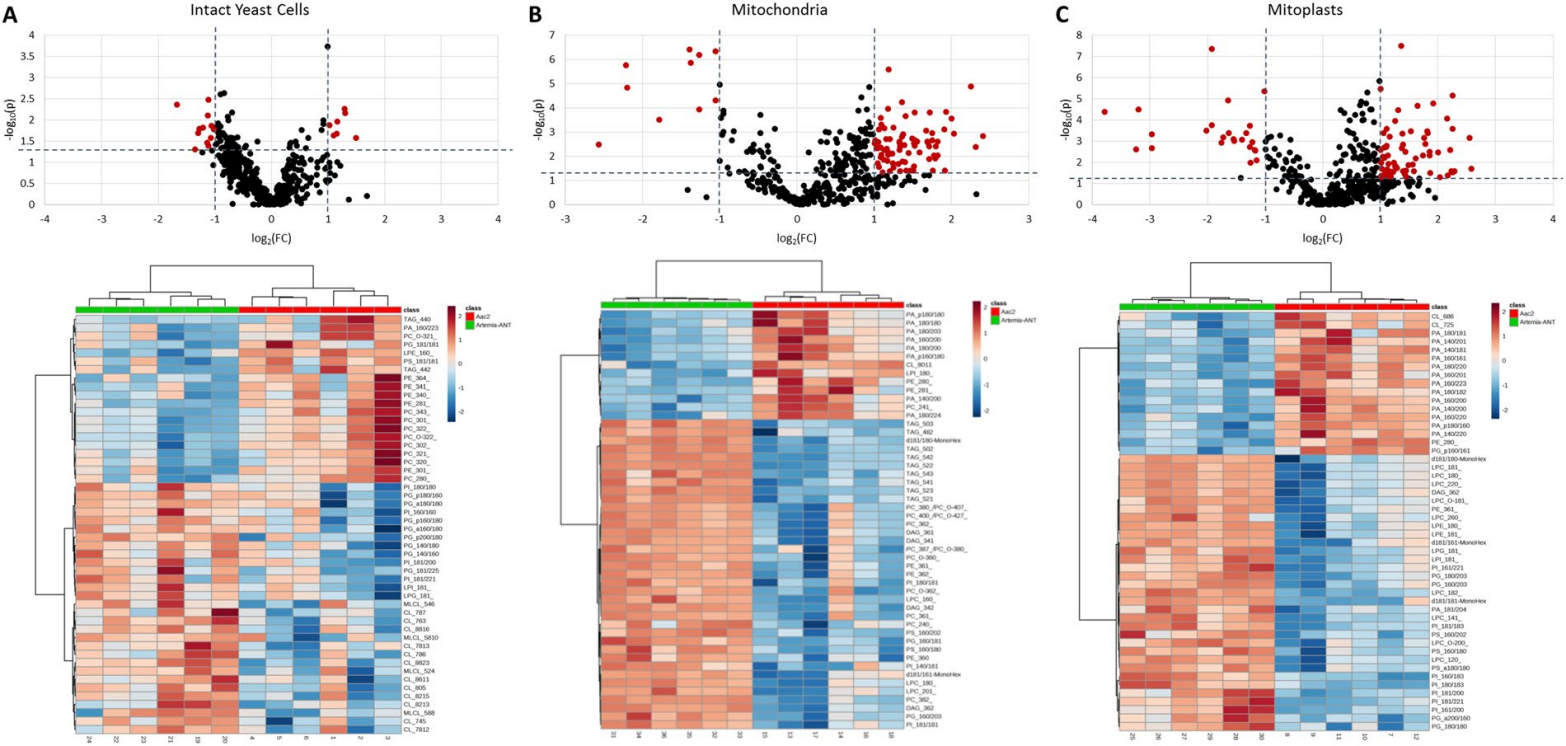
Volcano plots with fold change (FC) and p values of molecular lipid species; red indicates a fold change >2 and p value < 0.05; heat maps generated through unsupervised hierarchical clustering of the top 50 molecular lipid species by ANOVA of (**A**) intact yeast cells, (**B**) mitochondria, and (**C**) mitoplasts of control yeast (Aac2) compared to ArANT expressing yeast performed by MetaboAnalyst 3.0.

To assess how the heterologous expression of ArANT in yeast affected the mitochondrial lipidome, we analyzed Aac2 and ArANT expressing intact yeast cells, mitochondria, and mitoplasts using a high resolution direct infusion MS/MS^ALL^ lipidomics platform. Over five hundred molecular lipid species from 18 different lipid classes were identified and quantified in all sample types (Table 1) using an in house database and internal standard cocktail. The data was filtered based on predetermined limits of detection for the platform. Lipidomics assessment demonstrated quantitative differences in mitochondria and mitoplast lipidomes upon heterologous expression of ArANT. Nineteen lipid molecular species changed significantly between Aac2 and ArANT expressing yeast, with p values ≤ 0.05 and fold change (FC) above 2 (Fig. 1A). That number increases to 90 statistically significant lipid molecular species when compared to isolated mitochondria (Fig. 1B), and increases further to 101 lipid molecular species in isolated mitoplasts (Fig. 1C).

**Table 1:** Molecular lipid species identified in Aac2- and ArANT yeast cells, mitochondria and mitoplasts.

Lipid Class	Aac2			ArANT		
	Intact Yeast Cell	Mitochondria	Mitoplasts	Intact Yeast Cell	Mitochondria	Mitoplasts
Cer	0	0	0	0	1	1
CE	2	2	2	2	2	2
DAG	16	16	16	16	16	16
GLY	1	3	2	0	4	4
LPA	4	4	4	4	4	4
PA	61	62	62	61	61	62
LPC	21	21	22	21	22	22
PC	45	46	46	45	46	46
LPE	4	5	5	5	5	5
PE	18	23	23	17	23	23
LPG	6	6	6	6	5	6
PG	98	101	100	101	101	101
LPI	5	5	5	5	5	5
PI	55	58	58	57	58	58
LPS	8	8	8	8	8	7
PS	37	37	37	37	37	37
TAG	47	49	48	48	49	49
CL	141	140	140	141	139	140
**TOTAL**	**568**	**585**	**583**	**572**	**585**	**586**

**Figure 2:**
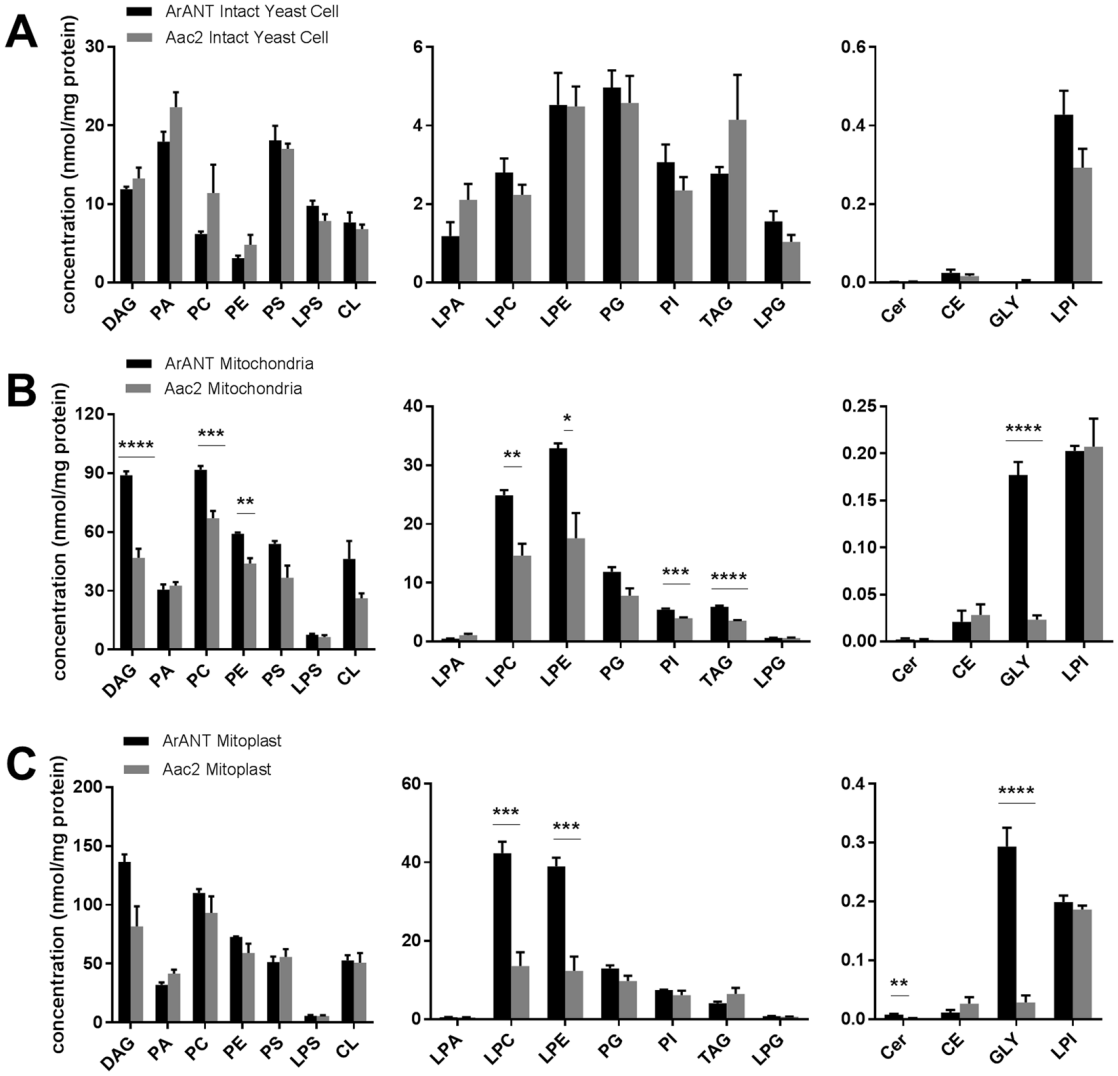
Concentration (nmol/mg protein) of lipid classes in control (Aac2) and *Artemia*-ANT expressing yeast cells (**A**), mitochondria (**B**), and mitoplasts (**C**). P values were calculated using multiple t-tests and statistical significance was determined using the Holm-Sidak method, with alpha = 0.05; *p ≤ 0.05, **p ≤ 0.01, ***p ≤ 0.001, ****p ≤ 0.0001. CE: cholesteryl ester; Cer: ceramide; CL: cardiolipin; DAG: diacylglycerol; GLY: glycolipid; PA: phosphatidic acid; LPA: lysophosphatidic acid; PC: phosphatidylcholine; LPC: lysophosphatidylcholine; PE: phosphatidylethanolamine; LPE: lysophosphatidylethanolamine; PG: phosphatidylglycerol; LPG: lysophosphatidylglycerol; PI: phosphatidylinositol; LPI: lysophosphatidylinositol; PS: phosphatidylserine; LPS: lysophosphatidylserine; TAG: triacylglycerol. Data shown are Mean +/− S.E.M. (n = 6).

Additionally, the concentration (nmol/mg protein) of lipids increases in mitochondria and mitoplasts compared to intact Aac2 and ArANT expressed yeast cells (Fig. 2). While there are no significant differences in concentration of lipid species in intact Aac2 and ArANT expressed yeast cells, there are significant changes in DAG, PC, LPC, LPE, PI, TAG, and GLY lipid classes in the respective mitochondria (Fig. 2B). Furthermore, there are even greater differences in LPC, LPE, and GLY lipid classes in Aac2 and ArANT expressed yeast mitoplasts. Concentrations (nmol/mg protein) for all molecular lipid species can be found in Supplementary Tables 1–4. Criteria for lipid identification for each lipid class include MS mode, molecular ion, scan mode, and fragment (m/z) are shown in Supplementary Table 5.

### Heterologous expression of ArANT in yeasts affects the function of other membrane-embedded proteins

**Figure 3:**
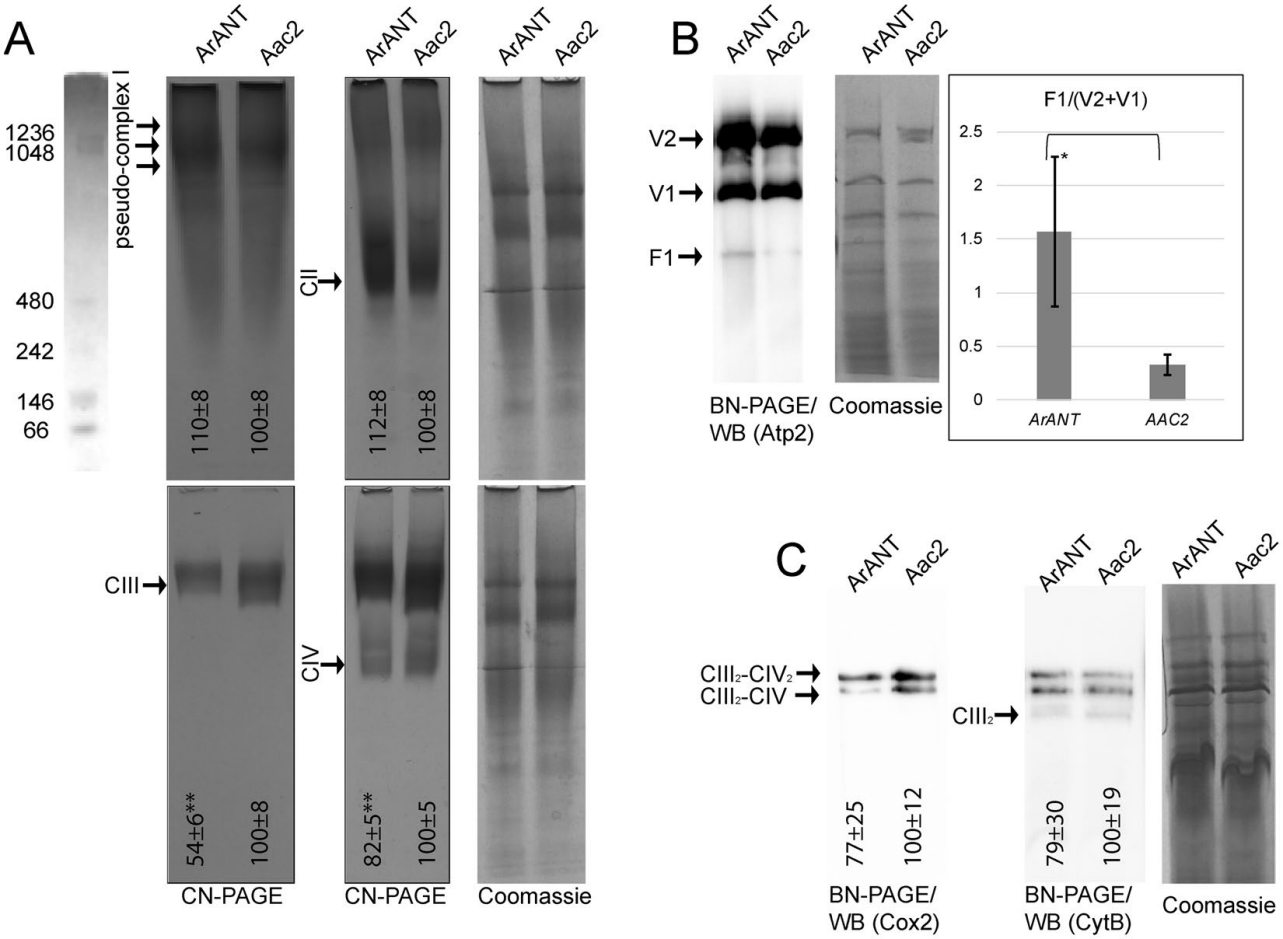
(**A**) In-gel enzymatic activities of mitochondrial respiratory chain complexes. Solubilized mitochondrial complexes (400 µg) were separated by clear-native polyacrylamide gel electrophoresis (CN-PAGE). Gels were stained with Coomassie blue to visualize the protein complexes, or incubated with appropriate reagents (see Materials and Methods) to visualize the activities of NADH dehydrogenases Nde1, Nde2 and Ndi1 (collectively referred to as pseudo-complex I), succinate dehydrogenase (CII), cytochrome c reductase (CIII) and cytochrome c oxidase (CIV). Activity products were imaged using a standard flatbed scanner. The gel-lane scan on the top-most left corner is a molecular weight ladder (NativeMark™ Unstained Protein Standard, Thermofisher Sci, imaged at a different contrast and saturation level), to which all gel scans shown in panel A can be compared to. Gel scan corresponding to pseudo-complex I was taken after 1 hour of exposure; a time-lapse of exposures (also for complex II) is shown in Supplementary Figure 1). (**B**) Blue native-PAGE followed by Western blot analysis of F_O_-F_1_ ATP synthase in dimers (V2), monomers (V1), and free catalytic head form (F1). (**C**) BN-PAGE analysis followed by Western blotting of complexes III and IV. The complexes were detected by using antibodies against CytB (for complex III) and Cox2 (for CIV)). For loading controls, gels shown in A, B and C were stained with Coomassie (three replicates); a representative gel is depicted on the right of each panel. Densitometric analysis of the gel bands shown in panels (A and C) are arbitrarily normalized to the signals obtained from Aac2 samples. Densitometric analysis of the gel bands shown in the rightmost panel (B) is represented as the ratio of free F1/(dimers + monomers). Data shown are Mean +/− S.E.M. (n = 5). For panel (C), the Densitometric analysis included all bands. CIII_2_-CIV_2_ implies supramolecular forms of two complex III and two complex IV entities; CIII_2_-CIV implies supramolecular forms of two complex III and one complex IV entity, while CIII_2_ implies supramolecular forms of two complex III entities. Images are representative of scanned gels from three to five independent experiments. *p < 0.05, and **p < 0.001.

In order to examine the effect of heterologous expression of ArANT in yeasts and how it affects the properties of other membrane-embedded proteins, we investigated in-gel and enzymatic activities of ETC complexes and probed for the presence of several key subunits. We further measured oxygen consumption rates in isolated mitochondria and quantitated ATP synthesis capacity and ATP hydrolytic activity of the F_O_-F_1_ ATP synthase complex. Viability of yeast strains, mitochondrial membrane potential, insertion of ArANT to inner mitochondrial membrane, ArANT primary structure verification and functionality have all been addressed in^31^. In-gel activities of ETC complexes are shown in Fig. 3. No significant differences were found for the in-gel activities of Nde1, Nde2 and Ndi1 (belonging to pseudo-complex I) and of complex II among the ArANT-expressing vs Aac2-expressing yeast mitochondria, normalized to the amount of loaded protein deduced by Coomassie staining shown in upper-right panel A (see numbers indicated within upper-left and upper-mid panel A). Although the bands corresponding to pseudo-complex I seem diffuse, this is not unexpected as several of its subunits bind to other subunits of the respiratory chain forming supercomplexes in yeasts^32,33^; thus, pseudo-complex I activity will be dispersed as a function of binding of its components to other subunits with a variable molecular weight. Lower in-gel complex III and IV activities were observed in the ArANT-expressing strain, compared to the Aac2-expressing strain (normalized to the amount of loaded protein deduced by Coomassie staining, bottom-right panel A). F_O_-F_1_ ATP synthase complex did not show any alterations in either dimeric (V2) or monomeric (V1) form in BN-PAGE electrophoresis, normalized by Coomassie staining. However, there was a higher level in the free F_1_ domain in the ArANT-expressing mitochondria, compared to those harboring Aac2 instead, depicted in upper-right panel B. The decrease of the in-gel complex III and IV activity were further investigated by probing for CytB (a complex III component) and Cox2 (a complex IV component by Western blotting after BN-PAGE (performed independently). No significant differences were found by comparing the density of bands obtained from ArANT-expressing strain vs the Aac2-expressing strain, normalized to the amount of loaded protein deduced by Coomassie staining, bottom-right panel C. The results shown in figure panel 3C imply that there were no appreciable differences in complex III and IV levels. It is to be noted that the lack of finding differences among complexes from the in-gel assays is limited by the methods used hereby; more sophisticated methods for addressing in-gel activities of mitochondrial complexes have been published elsewhere^34^. Finally, we must also stress that our experiments did not include measurements of transcript levels of ETC components as performed by Nůsková *et al*.^30^, thus it cannot be excluded that changes observed hereby were mediated on a genetic level.

**Figure 4:**
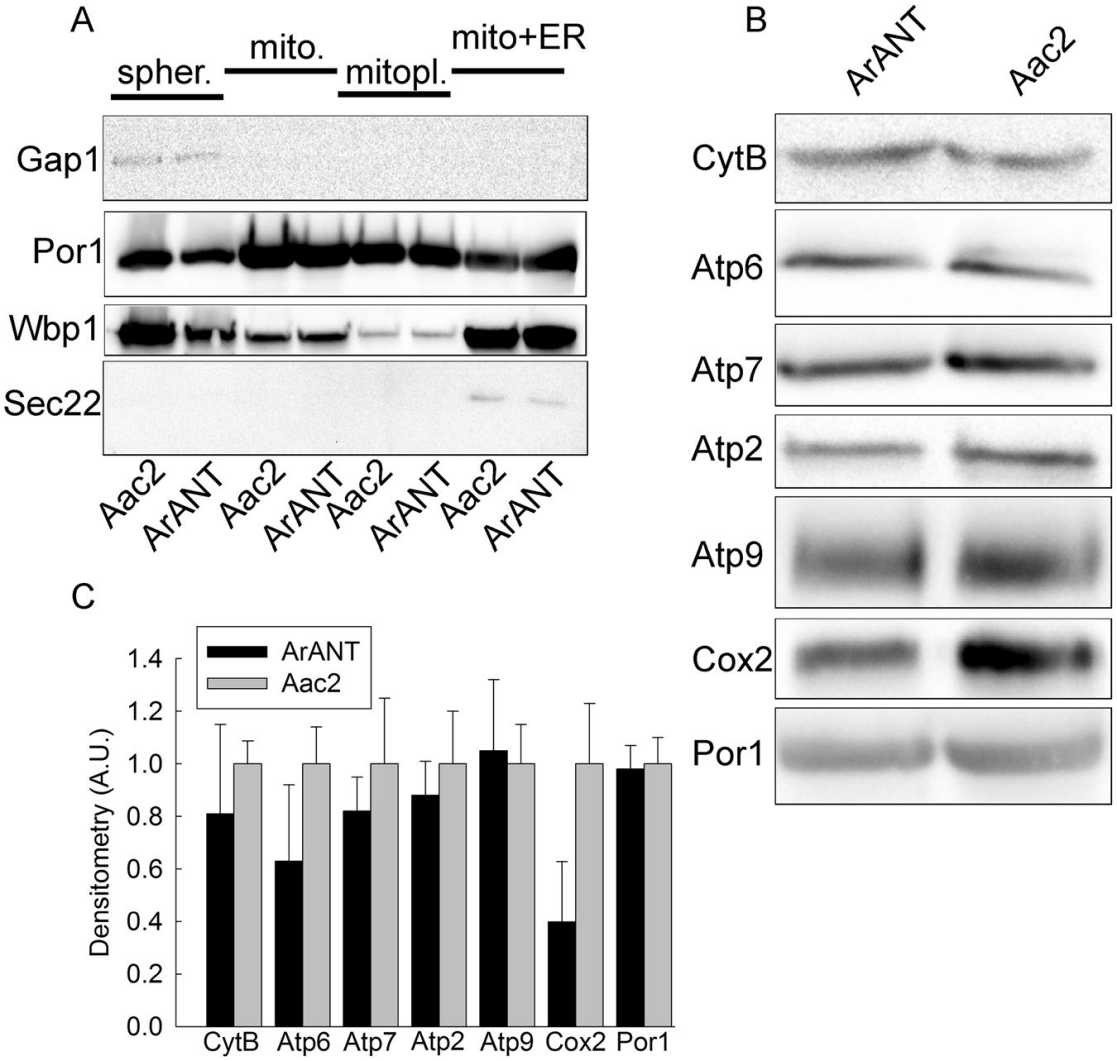
(**A**) Scanned images of Western blotting comparing the expression of proteins indicated on the left of each panel from yeasts expressing ArANT *vs* Aac2. Spher.: spheroplasts; mito.: mitochondria; mitopl.: mitoplasts; mito + ER: the fraction located between the 15% and 23% of Percoll during mitochondrial purification, containing ER, Golgi and other organelles. (**B**) Scanned images of Western blotting comparing the expression of mitochondrial proteins indicated on the left of each panel, from yeasts expressing ArANT *vs* Aac2. (**C**) Densitometric analysis of the bands pooled from three gels produced as shown in panel (B), arbitrarily normalized to the signals obtained from Aac2 samples. Data shown are Mean +/− S.E.M. (n = 3). A.U.: arbitrary units. All images are representative from three independent experiments.

To address the possibility that mitochondria and mitoplast fractions obtained from the ArANT and AAC2-expressing strains exhibited a different degree of contamination from non-mitochondrial components potentially affecting lipidomic measurements, we probed for the expression of plasma membrane (Gap1), ER (Wbp1) and ER-Golgi (Sec22) markers. As shown in Fig. 4A, yeast cells (spheroplasts: spher.), mitochondria (mito.), mitoplasts (mitopl.), and the mitochondria plus ER fraction obtained during the Percoll protocol (mito + ER) were probed for the aforementioned proteins by standard Western blotting. The plasma membrane marker Gap1 appeared only in the spheroplasts, while the ER-Golgi marker Sec22 was evident only in the mito + ER fractions. Although it should have also been present in the spheroplasts fraction, there it was absent probably due to the fact that it yields a weak antibody-antigen interaction (‘weak band’). The extent of contamination by ER in the various fractions is better appreciated by inspecting the Wbp1 blot: there, it is apparent that the extent of ER contamination is largely diminished upon mitochondria and mitoplast purification, and there is no difference between those obtained from the ArANT- vs Aac2-expressing yeasts. The WB of Por1 confirms the extent of enrichment of mitochondrial outer membranes in the various fractions. Although porin is an exclusive component of the outer mitochondrial membrane, the association of this protein with the so-called ‘contact sites’^35^, parts where the outer and inner mitochondrial membranes exhibit strong affinity, mitoplast fractions depleted from outer membrane still stain positive for porin, as it has been shown elsewhere^36^. From the results shown in Fig. 4A we concluded that if there is differential contamination of mitochondrial/mitoplast fractions by ER components, it is below the detection limit of the method used.

To investigate further the effect of heterologous expression of ArANT in yeasts, we probed for several key membrane-embedded ETC components, specifically CytB, Atp6, Atp7, Atp2, Atp9, Cox2, and normalized them to the expression level of porin (Por1: isoform 1) by Western blotting. As shown in Fig. 4B, except Cox2 (a subunit of complex IV) there were no other alterations in the contents of the probed membrane-embedded subunits. Densitometric analysis of the bands pooled from three replicate gels are shown in Fig. 4C. No statistical significance was observed by comparing band densities for any protein tested between ArANT (black bars) and Aac2 (grey bars) yeast samples.

**Figure 5:**
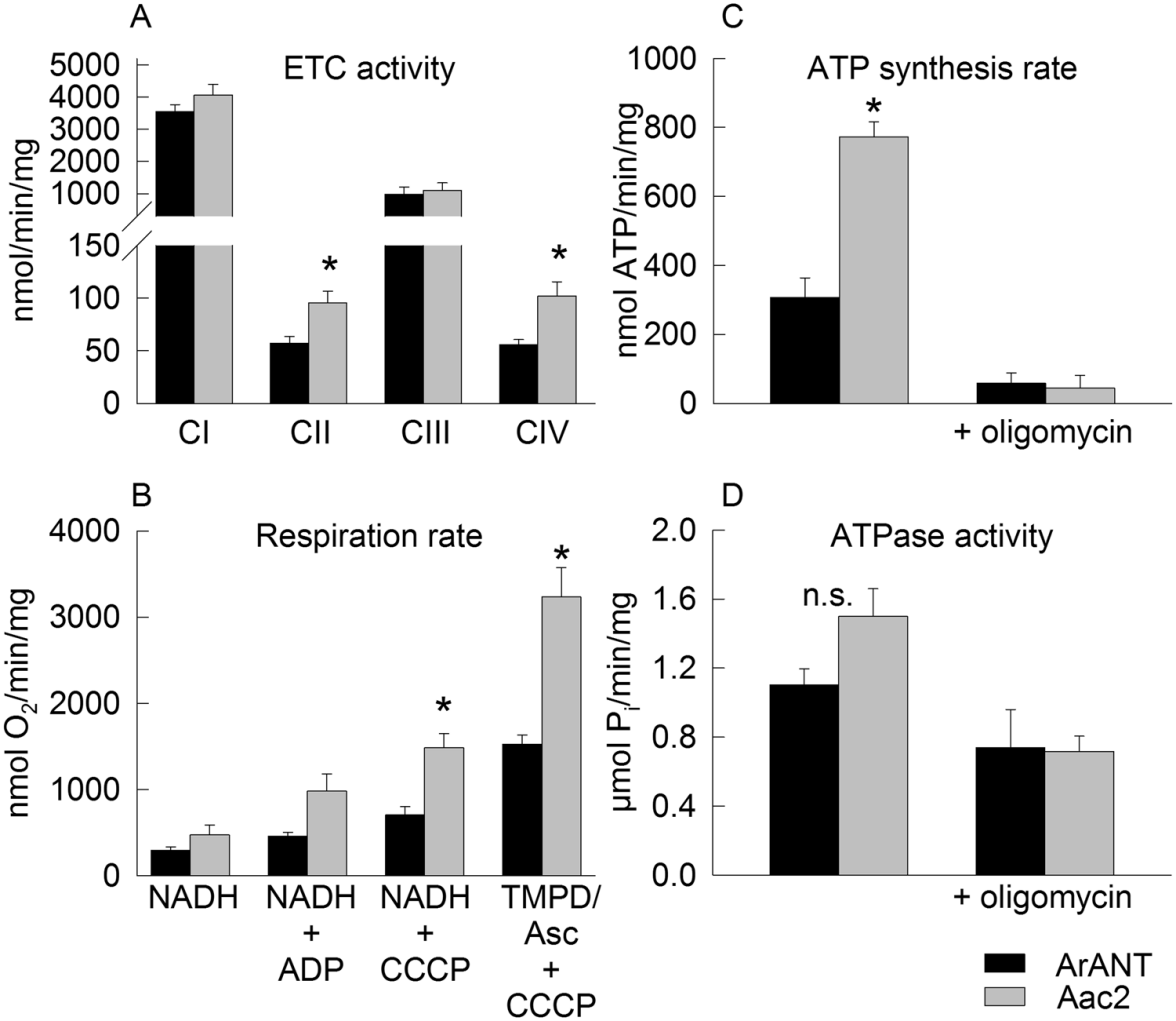
Bioenergetic profiling of ArANT- (black) and Aac2- (grey) expressing yeast mitochondria. (**A**) Enzymatic activities of respiratory chain complexes. CI activity is attributed to rotenone-insensitive Nde1, Nde2 and Ndi1 NADH dehydrogenases. (**B**) Respiration rates of isolated mitochondria, measured after the consecutive additions of 4 mM NADH (state 2 respiration), 150 µM ADP (state 3), 4 µM carbonyl cyanide m-chlorophenylhydrazone (CCCP) (uncoupled respiration), or CCCP + ascorbate + TMPD (exclusive complex IV-dependent respiration). (**C,D**) ATP synthesis and hydrolysis rates of isolated mitochondria. The rates of ATP synthesis were determined using 4 mM NADH and 750 µM ADP, in the presence or absence of 3 µM oligomycin, as indicated in the panels. ATPase activities were measured in the absence of osmotic protection, at pH 8.4. All data shown are Mean +/− S.E.M. (n = 3). For panels (A–C), p values were calculated using t-tests; *p ≤ 0.05.

To evaluate the impact of alterations conferred by the heterologous expression of ArANT observed in the BN-, CN- and SDS-PAGE experiments on mitochondrial functionality, we measured individual complex activities, oxygen consumption during various metabolic conditions, and ATP synthesis and hydrolysis rates. As shown in figure panel 5A, enzymatic activities of complexes II and IV were significantly decreased in mitochondria expressing ArANT, comparing to those harboring Aac2. The decrease in complex IV activity was also reflected in the significantly decreased rate of oxygen consumption when TMPD + ascorbate −obligate substrates of exclusively complex IV- were used, in ArANT expressing mitochondria (figure panel 5B). State 2 and 3 respiration did not reach statistical significance between the yeast strains, even though ArANT mitochondria exhibited a ~2-fold decrease in state 3 respiration compared to that obtained by Aac2-harboring mitochondria. Subsequent addition of the uncoupler CCCP though afforded a statistical significance in the comparison of respiration rates between ArANT- and Aac2-harboring mitochondria. Regarding ATP synthesis/hydrolysis rates mediated by F_O_ -F_1_ ATP synthase, results are shown in figure panels 5 C and 5D, respectively. The rate of ATP synthesis (measured at state 3 with NADH as a respiratory substrate) was ~60% lower in the ArANT-expressing than Aac2-expressing mitochondria. The lack of statistical significance between ArANT and Aac2-expressing mitochondria during state 3 respiration shown in Fig. 5B may seem at odds with the finding that ArANT-expressing mitochondria produce less amount of ATP per unit time, depicted in Fig. 5C; however, it is important to consider that the rate of state 3 respiration and rate of ATP production need not be in a linear relationship. It may well be possible that the flux-control coefficient of F_O_-F_1_ ATP synthase working in the forward mode is not sufficiently high to warrant a strong influence in state 3 respiration. The rate of mitochondrial ATP hydrolysis was assessed at pH = 8.4 in non-osmotically protected conditions and in the presence of saturating amounts of ATP aiming for maximal ATPase activity rates; alkaline pH prevents the binding of IF1 to ATP synthase^37^, and ATP hydrolytic activity is not limited by a proton gradient. ATPase activity in ArANT-expressing mitochondria was reduced by 25% compared to the Aac2-harboring mitochondria, and free F1 likely mediated 67% of this activity since it was only partially sensitive to oligomycin. However, as a word of caution, the oligomycin-insensitive ATP hydrolysis rate could also be due to non-mitochondrial ATPases contaminating the mitochondrial fractions. The extent of this contamination cannot be reliably quantified, however, on the basis of the results shown in Fig. 4 it is anticipated that it is similar between ArANT- and Aac2-expressing mitochondria. In Aac2-harboring mitochondria, approximately 47% of ATPase activity was mediated by free F1 plus contaminating non-mitochondrial ATPases.

## Discussion

In the present study we investigated the effect of heterologous expression of ArANT in yeast by examining their total, mitochondrial and mitoplastic lipidomes and interrogating the function of other proteins embedded in the same membrane as the heterologously expressed one. Our results demonstrated that there were both qualitative and quantitative lipidomic changes conferred by expressing ArANT in the inner mitochondrial membrane. Most notably, diacylglycerols, phosphatidylcholines, lysophosphatidylcholines, lysophosphatidylethanolamines, phosphatidylinositols, triacylglycerols and glycolipids were all significantly increased in the mitochondrial membranes of ArANT expressing yeasts. These changes suggest significant biophysical adaption of the subcellular mitochondrial membrane as well as inner mitochondrial membrane in response to metabolic activity. This is evidenced by lysolipid formation as well as neutral lipid and glycerol-lipid content changes. In addition to the lipidomic changes, heterologous expression of ArANT also affected the enzymatic activities of complexes II, IV and the F_O_ − F_1_ ATP synthase. In-gel assays of complex II did not show a difference between the ArANT and Aac2-harboring yeast strains, however, these assays are not as sensitive as the enzymatic ones.

It is to be noted that neither composition nor the amount of cardiolipins were different between the ArANT- and Aac2 expressing yeast mitoplasts. This is important because cardiolipins are essential for the optimum activity of many inner mitochondrial membrane proteins, including the translocase itself^38–43^. It can be therefore stated that the alterations in mitochondrial lipidome caused by the heterologous expression of ArANT affecting the functions of other membrane-embedded proteins was not due to a catastrophic change in cardiolipins composition, in line with the biochemical stability and overall viability shown for this yeast strain^31^.

Our work outlines two ramifications: firstly, by heterologously expressing a membrane-embedded protein in a host, the membrane lipidome is altered. It cannot be overemphasized that our work concerns an individual case, that of expressing ArANT in the yeast strain MWY83/1; However, it sets the precedent of checking for changes in the membrane lipidome in experiments designed to include heterologous expression of other membrane-embedded proteins in different hosts. It is presently not possible to decipher how ArANT expression might perturb the mitochondrial lipidome. This problem is further confounded by the so-called ‘ripple effect’, whereby a change in the level of one lipid indirectly induces changes in the levels of other lipids^44^. Thus, a ‘reductionist approach’ would probably be an oversimplification. A loss or decrease of one lipid class may have no effect, and the interaction of loss or decrease of two or more lipid classes may actually be responsible for the alteration. Also, research regarding lipids is lagging compared to research regarding proteins and nucleic acids. Systematic investigation is needed on the mechanisms by which lipids modulate protein function and structure^45^. Relevant to this, specialized protocols involving liposome-microarray-based assays (LIMA) are just being developed^46^.

Secondly, the alterations in membrane lipidome conferred by the heterologous expression of a foreign protein, in turn, may alter the function of other membrane-embedded proteins. The concept of lipid modulation of membrane-embedded proteins is now becoming recognized^15,19,47–49^, at least to some extent^50^. Furthermore, it has been proposed that lipids can act as “lipochaperones” thus influencing protein folding in diseases such as Alzheimer’s, prion/scrapie disease, cystic fibrosis, cataract formation, and type 2 diabetes^43^. It must be emphasized that protein-protein interactions may also be responsible for changes observed in view of the fact that membrane lipid composition alterations can affect membrane-embedded proteins. Specifically, it is known that overexpressing a protein in a host may reduce the steady-state levels of other proteins, or disrupt stoichiometric complexes, compete for a shared subunit of two complexes or sequester a protein, reviewed in^1^. At least for human adenine nucleotide translocases It has been recently shown that they physically and functionally interact with the respirasome, *i.e*., membrane-embedded subunits of the electron transport chain^51^. Furthermore, in yeast it has been demonstrated that the synthesis of cytochrome c oxidase subunit 1 is downregulated in the absence of a functional F_O_-F_1_-ATP synthase, through a mechanism dependent on Cox1p synthesis^52^. Thus, it is impossible to decipher the extent of contribution of lipidome changes on affecting membrane-embedded functions vs that due to direct protein-protein interactions. Further studies will be needed to elucidate the mechanism(s) by which heterologously expressed proteins alter host lipidome and associated membrane proteins.

## Materials and Methods

### Expression of ArANT in yeast cells

The yeast strains used in this study were MWY83/1 (ArANT, nuclear phenotype: *MATa ade2-1 his3-11,15 trp1-1 leu2-3,112 ura3-1 arg8Δ::HIS3 aac1Δ::KanMX4 aac3Δ::KanMX4 aac2Δ::ArAAC-HAHphNTI sal1Δ::NatMX4* [*AAC2*/pYep352]) and MWY84/3 (*AAC2*, nuclear phenotype: *MATa ade2-1 his3-11,15 trp1-1 leu2-3,112 ura3-1 arg8Δ::HIS3 aac1Δ::KanMX4 aac3Δ::KanMX4 sal1Δ::NatMX4*) were constructed exactly as described in^31^. In strain MWY83/1, ArANT is under the *AAC2* promoter which is integrated in the genome also expressing AAC2 driven by its own promoter from a co-inserted multi-copy plasmid, while strain MWY84/3 expresses only isoform *AAC2*.

### Isolation and bioenergetic analyses of yeast mitochondria

Assays were performed on mitochondria isolated from yeast cells grown in rich galactose medium (YPGalA, 1% Bacto yeast extract, 1% Bacto Peptone, 2% galactose, 40 mg/l adenine) at 28 °C. The mitochondria were isolated as previously described^53^. Oxygen consumption rates were measured with a Clark electrode in a buffer consisting of 0.65 M mannitol, 0.36 mM EGTA, 5 mM Tris-phosphate, 10 mM Tris/maleate, pH 6.8, as previously described^54^. For ATP synthesis rate measurements, the mitochondria (0.15 mg/ml) were placed in a 1 ml thermostatically controlled chamber at 28 °C in the same buffer as for the polarographic measurements. The reaction was started by adding 4 mM NADH and 0.15 mM ADP (for respiration assays) or 0.75 mM ATP (in the presence or absence of oligomycin, for determination of ATP synthesis or hydrolysis rates). 100 µl aliquots were taken either every 15 seconds and the reaction was stopped by adding 3.5% perchloric acid and 12.5 mM EDTA. Samples were neutralized to pH 6.5 by KOH and 0.3 M MOPS. ATP was quantified by Kinase-Glo Max Luminescence Kinase Assays (Promega) using a Beckman Coulter’s Paradigm Plate Reader. Other additions were 12.5 mM ascorbate, 1.4 mM N,N,N,N,-tetramethyl-p-phenylenediamine (TMPD), 4 μM CCCP or 3 μg/ml oligomycin, where indicated. The specific ATPase activity was measured at pH 8.4 using a previously described procedure, quantitating the amount of liberated phosphate from ATP hydrolysis^55^.

Electron transport chain complex and citrate synthase activity assays: Enzymatic activities of Complex I (attributed to rotenone-insensitive Nde1, Nde2 and Ndi1)^56,57^, complex II, complex III and complex IV were determined in isolated mitochondria as previously described^58–60^.

### Blue native, clear native and SDS-PAGE analyses

Blue native (BN)-PAGE or clear native (CN)-PAGE experiments were carried out as previously described^61^; briefly, 400 µg of mitochondrial proteins were suspended in 100 µl of extraction buffer (30 mM HEPES, 150 mM potassium acetate, 12% glycerol, 2 mM 6-aminocaproic acid, 1 mM EGTA, 2% digitonin (BN-PAGE) or 10% digitonin (BN- or CN-PAGE), protease inhibitor cocktail tablet (Roche) and 1 mM PMSF. After 30 min incubation on ice, the extracts were cleared by centrifugation (14,000 rpm, 4 °C, 30 min) and supplemented with 4.5 µl of loading dye (5% Serva Blue G-250, 750 mM 6-aminocaproic acid for BN-PAGE or 750 mM 6-aminocaproic acid for CN-PAGE) and 40 µl were loaded onto the gels electrophoresis. No detergents were present in either BN- or CN-PAGE electrophoresis. The CN-PAGE migration buffer composition was: 50 mM Bis-Tris and 50 mM tricine, pH 7.5. The same buffer was used for BN-PAGE as the anode buffer, and the cathode buffer was further supplemented with 0.02% Coomassie Blue G-250. Gels were run at room temperature at a constant voltage of 150 V. For SDS-PAGE analysis, mitochondrial proteins were suspended in Laemmli sample buffer at a concentration of 2 µg/µl and 50 µg of proteins were loaded per lane of a 12% SDS-PAGE gel and transferred onto nitrocellulose membranes. BN-Page gels were transferred onto PVDF membranes. Proteins were detected by polyclonal antibodies raised against ATP synthase subunits Atp2 (β), Atp7 (d), and Atp9 (c) at 1:10,000 dilution (gifts from Dr. M.F. Giraud, Bordeaux). Anti-CytB antibody (gift from prof. J.P. di Rago) and anti-Cox2 (Thermo Fisher) were used in titer of 1:5,000.

### In-gel catalytic activities assays

Regarding NADH dehydrogenases and complex II, CN-PAGE gels were washed with 5 mM Tris-HCl pH 7.4. For NADH dehydrogenases, gel was incubated in a buffer composed of 5 mM Tris-HCl pH 7.4, 1 mg/ml NBT and 0.2 mM NADH until blue strips appeared and photographed. For complex II assay, gels were washed in 5 mM Tris-HCl pH 7.4 several times and incubated in a buffer composed of 5 mM Tris-HCl pH 7.4, 1 mg/ml NBT, 20 mM sodium succinate and 0.2 mM PMS) until blue/violet strips appeared and photographed. Regarding complexes III and IV, CN-PAGE gels were washed with 5 mM Tris-HCl pH 7.4. For complex III activity, gels were incubated in a buffer composed of 5 mM Tris-HCl pH 7.4 and 0.5 mg/ml DAB until brown strips appeared and photographed. In gel activity assay for complex IV was performed in the same gels; gels were washed in 5 mM Tris-HCl pH 7.4 several times and incubated in a buffer consisting of 5 mM Tris-HCl pH 7.4, 0.5 mg/ml DAB and 0.05 mM cytochrome c until new brown strips appeared and photographed.

### Liquid/Liquid Extraction of Structural Lipids

Mitoplasts from Percoll-purified yeast mitochondria, Percoll-purified yeast mitochondria, and intact yeast cells were thawed and diluted with a ten-times diluted PBS solution. All samples were homogenized in Omni bead tubes with 2.8 mm ceramic beads in the Omni Bead Ruptor 24 with Cryo Cooling Unit (Omni International, Kennesaw, GA) at 4 °C for 2 minutes. Protein concentration was determined by the bicinchoninic acid assay and 1 mg of protein from mitoplast, mitochondria, and intact yeast cell samples were aliquoted and a cocktail of deuterium-labeled and odd chain phospholipid standards from diverse lipid classes was added. Standards were chosen to represent each lipid class and were at designated concentrations to provide the most accurate quantitation and dynamic range for each lipid species (Supplementary Table 5). To each sample, 4 mL chloroform:methanol (1:1, by vol) was added and lipidomic extractions were performed as previously described [Kiebish *et al*., 2010]. Lipid extraction was automated using a customized sequence on a Hamilton Robotics STARlet system (Hamilton, Reno, NV). Lipid extracts were dried under nitrogen and reconstituted in chloroform: methanol (1:1, by vol). Samples were flushed with nitrogen and stored at −20 °C.

### Direct Infusion MS/MS^ALL^ Structural Lipidomics Platform

Samples were diluted 50 times in isopropanol:methanol:acetonitrile:water (3:3:3:1, by vol.) with 2 mM ammonium acetate in order to optimize ionization efficiency in positive and negative modes. Electrospray ionization-MS was performed on a TripleTOF® 5600+ (SCIEX, Framingham, MA), coupled to a customized direct injection loop on an Ekspert microLC200 system (SCIEX). A sample volume of 50 µL was injected at a flow-rate of 6 µL/min. Lipids were analyzed using a customized data independent analysis strategy on the TripleTOF® 5600+ allowing for MS/MS^ALL^ high resolution and high mass accuracy analysis as previously described [Simons *et al*., 2012]. A customized in-house method was used for CL species with lower collision energy and shifted isolation windows. Quantification was performed using an in-house library on MultiQuant™ software (SCIEX). Heat maps generated through unsupervised hierarchical clustering of the top 50 molecular lipid species was performed by MetaboAnalyst 3.0^62^.

### Standards and Chemicals

Pre-cast gels were purchased from Thermo Fisher Scientific. NADH, tricine, lead (II) nitrate, nitrotetrazolium blue chloride, 3,3′-diaminobenzidine tetrahydrochloride hydrate, phenazine methosulfate, phenylmethylsulfonyl fluoride, cytochrome c (from bovine heart), and digitonin were from Sigma. Coomassie Blue G-250 was from Serva. Bis-Tris (Bis[2-hydroxyethyl] amino-tris[hydroxymethyl]-methane was from Bioshop. Bicine was from AppliChem. Protease inhibitor cocktail tablets were from Roche. Regarding lipidomics, all standards were purchased from Avanti Polar Lipids (Alabaster, AL), Nu-Chek Prep Inc. (Waterville, MN), Matreya (State College, PA), Cayman Chemical Company (Ann Arbor, MI), Sigma-Aldrich (St. Louis, MO), or Cambridge Isotope Laboratories (Tewksbury, MA). All solvents were of HPLC or LC/MS grade and were acquired from Fisher Scientific (Waltham, MA) or VWR International (Radnor, PA).

## Electronic supplementary material

Supplementary Tables 1-5 and Supplementary figure 1
