# Supplementary material for: Perturbation of the yeast mitochondrial lipidome and associated membrane proteins following heterologous expression of Artemia-ANT
Source: Sci Rep. 2018 Apr 12;8:5915. doi: 10.1038/s41598-018-24305-2 (PMC5897331; doi:10.1038/s41598-018-24305-2)
Supplement: Supplementary file 1 — Supplementary Tables 1-5 and Supplementary figure 1 [file 41598_2018_24305_MOESM1_ESM.pdf]

Perturbation of the yeast mitochondrial lipidome and associated membrane proteins following heterologous expression of Artemia-ANT

Emily Chen[1], Michael A. Kiebish[1], Justice McDaniel[1], Katarzyna Niedzwiecka[2], Roza Kucharczyk[2], Dora Ravasz[3,4], Fei Gao[1], Niven R. Narain[1], Rangaprasad Sarangarajan[1], Thomas N. Seyfried[5], Vera Adam-Vizi[3,6] and Christos Chinopoulos[*3,4]

[1]BERG LLC, Framingham, MA 01701, USA
[2]Institute of Biochemistry and Biophysics, Polish Academy of Sciences, Warsaw, 02-106, Poland
[3]Department of Medical Biochemistry, Semmelweis University, Budapest, 1094, Hungary
[4]MTA-SE Lendület Neurobiochemistry Research Group, Budapest, 1094, Hungary
[5]Biology Department, Boston College, Chestnut Hill, Boston, MA, 02467, USA
[6]MTA-SE Laboratory for Neurobiochemistry, Budapest, 1094, Hungary

*Address correspondence to: Dr. Christos Chinopoulos, Department of Medical Biochemistry, Semmelweis University, Budapest, Tuzolto street 37-47, 1094, Hungary. Tel: +361 4591500 ext. 60024, Fax: +361 2670031. E-mail: chinopoulos.christos@eok.sote.hu

Supplementary Table 1: Concentrations (in pmol/mg protein) of ceramides (Cer), glycolipids, lysophosphatidylcholines (LPC), phosphatidylcholines (PC), lysophosphatidylethanolamines (LPE), and phosphatidylethanolamines (PE) in Aac2 and ArANT expressing yeast cells, Percoll-purified mitochondria and mitoplasts. N.D.: not determined. B.Q.L.: below quantitative limit. O-(carbon:double bond) indicates a plasmalogen species. Results shown are Mean +/- S.E.M.

		Control									Artemia-ANT								
$[M+H]^+$	Species	Yeast Cell			Mitochondria			Mitoplasts			Yeast Cell			Mitochondria			Mitoplasts		
680.5	Cer_d18:0/26:0	B.Q.L.	±	B.Q.L.	N.D.	±	N.D.	B.Q.L.	±	B.Q.L.	B.Q.L.	±	B.Q.L.	4.973	±	0.6591	1.237	±	0.7837
648.5	Cer_d18:1/24:1	1.617	±	0.7236	1.034	±	0.654	1.941	±	0.8818	1.056	±	0.6678	2.822	±	0.9624	1.255	±	0.7954
698.5	d18:1/16:1-MonoHex	B.Q.L.	±	B.Q.L.	4.630	±	2.493	2.940	±	1.471	B.Q.L.	±	B.Q.L.	38.59	±	5.947	24.75	±	2.318
700.6	d18:1/16:0-MonoHex	B.Q.L.	±	B.Q.L.	B.Q.L.	±	B.Q.L.	B.Q.L.	±	B.Q.L.	B.Q.L.	±	B.Q.L.	6.386	±	0.9317	1.264	±	0.8042
726.6	d18:1/18:1-MonoHex	B.Q.L.	±	B.Q.L.	0.8924	±	0.8924	B.Q.L.	±	B.Q.L.	B.Q.L.	±	B.Q.L.	14.70	±	1.474	9.94	±	1.188
728.6	d18:1/18:0-MonoHex	2.996	±	2.996	22.97	±	9.343	20.52	±	3.265	B.Q.L.	±	B.Q.L.	233.9	±	25.04	141.2	±	10.56
412.3	LPC_10:0_	5.692	±	0.5157	3.422	±	0.8125	7.794	±	1.134	7.409	±	1.666	5.535	±	0.2296	6.613	±	0.2719
440.3	LPC_12:0_	20.13	±	2.037	16.06	±	3.866	44.61	±	4.108	12.99	±	0.8787	75.50	±	5.643	44.17	±	2.520
468.3	LPC_14:0_	12.90	±	1.310	71.30	±	18.81	78.39	±	7.839	8.075	±	1.374	153.2	±	9.927	112.7	±	7.225
466.3	LPC_14:1_	25.89	±	3.420	48.54	±	7.530	90.75	±	11.75	26.71	±	2.204	129.1	±	11.67	77.03	±	3.357
496.3	LPC_16:0_	132.2	±	19.12	612.1	±	146.5	529.0	±	53.53	111.4	±	10.30	1484	±	101.3	1002	±	45.86
494.3	LPC_16:1_	554.4	±	71.33	6401	±	1616	7029	±	738.9	585.4	±	80.38	17330	±	1239	9924	±	433.9
524.4	LPC_18:0_	158.4	±	19.59	374.0	±	101.8	346.7	±	48.75	173.4	±	11.55	1172	±	63.75	752.6	±	24.84
522.4	LPC_18:1_	1191	±	178.4	5730	±	1558	6194	±	1148	1740	±	253.5	21050	±	1492	12490	±	453.7
520.4	LPC_18:2_	5.423	±	0.4894	46.58	±	8.057	46.78	±	3.313	9.765	±	1.032	234.6	±	16.72	49.36	±	4.037
552.4	LPC_20:0_	7.015	±	0.8555	44.30	±	12.67	58.79	±	6.040	6.821	±	1.030	123.3	±	13.10	63.74	±	5.395
550.4	LPC_20:1_	15.46	±	1.849	22.84	±	7.552	21.49	±	3.872	19.95	±	2.757	69.25	±	4.300	56.51	±	4.128
580.4	LPC_22:0_	10.07	±	1.318	37.03	±	10.96	44.63	±	6.450	12.67	±	2.031	146.0	±	13.12	74.24	±	3.126
608.5	LPC_24:0_	10.66	±	3.959	34.70	±	9.034	31.17	±	9.574	10.35	±	1.946	76.15	±	8.753	60.64	±	2.661
606.5	LPC_24:1_	11.57	±	2.669	6.565	±	1.027	8.128	±	0.7708	10.84	±	1.432	7.674	±	0.772	6.876	±	0.9144
636.5	LPC_26:0_	28.32	±	6.765	31.01	±	4.990	26.23	±	1.917	21.59	±	1.736	53.38	±	4.499	40.16	±	2.130
634.5	LPC_26:1_	12.18	±	2.283	17.22	±	2.028	15.84	±	0.9973	12.31	±	1.959	24.89	±	1.632	17.8	±	1.207
482.3	LPC_O-16:0_	2.238	±	1.049	7.132	±	1.930	6.475	±	1.144	1.408	±	0.8909	13.40	±	1.431	10.00	±	1.003
480.3	LPC_O-16:1_	2.653	±	0.5424	20.29	±	5.257	27.14	±	3.187	2.770	±	0.9158	54.42	±	4.737	29.42	±	2.634
508.4	LPC_O-18:1_	6.135	±	0.9835	27.46	±	5.691	28.72	±	2.186	6.359	±	0.8757	65.48	±	2.894	38.81	±	3.318
538.4	LPC_O-20:0_	7.927	±	0.6309	8.194	±	2.005	15.89	±	3.418	10.69	±	1.43	24.26	±	2.795	12.83	±	0.6715
536.4	LPC_O-20:1_	1.790	±	0.8754	4.245	±	1.161	5.143	±	0.7835	3.432	±	1.368	9.655	±	0.8034	5.064	±	1.130
566.4	LPC_O-22:0_	9.112	±	1.752	7.415	±	0.6122	10.35	±	2.207	7.193	±	0.9901	9.856	±	0.8282	14.55	±	1.784
622.5	PC_24:0_	18.00	±	2.426	24.95	±	5.051	19.48	±	2.441	15.03	±	1.645	38.66	±	3.723	32.25	±	1.705
620.5	PC_24:1_	182.4	±	12.57	28.57	±	3.876	44.27	±	3.010	168.7	±	26.88	22.08	±	2.003	26.96	±	1.643
650.5	PC_26:0_	74.83	±	15.23	210.3	±	28.30	144.5	±	14.02	48.52	±	3.888	177.2	±	11.61	159.3	±	7.344

648.5	PC_26:1_	1025	±	49.68	1116	±	47.42	1105	±	61.79	858.3	±	38.78	1012	±	30.49	960.1	±	16.76
678.5	PC_28:0_	107.7	±	25.06	576.4	±	81.22	418.6	±	40.63	64.45	±	2.834	555.3	±	34.58	439.7	±	17.62
676.5	PC_28:1_	1801	±	91.25	3938	±	372.2	3170	±	173.3	1536	±	39.34	3378	±	142.3	2958	±	56.51
706.6	PC_30:0_	59.21	±	16.88	405.0	±	55.54	292.4	±	29.91	29.28	±	3.563	402.8	±	20.79	326.2	±	11.71
704.6	PC_30:1_	234.0	±	88.37	3066	±	472.7	2034	±	184.1	85.84	±	5.805	2577	±	162.7	1999	±	43.17
702.6	PC_30:2_	86.17	±	25.82	1336	±	258.0	788.1	±	42.68	38.76	±	3.598	961.1	±	50.96	759.2	±	12.03
700.6	PC_30:3_	3.121	±	1.169	23.04	±	2.975	17.50	±	1.414	0.9782	±	0.6187	21.35	±	1.822	18.80	±	1.291
734.6	PC_32:0_	87.33	±	37.55	925.8	±	148.0	640.8	±	69.96	35.14	±	2.436	1023	±	60.65	773.7	±	27.15
732.6	PC_32:1_	1140	±	454.8	9827	±	1297	7167	±	382.3	429.1	±	29.83	10590	±	274.0	8713	±	170.5
730.6	PC_32:2_	2312	±	922.4	21450	±	2427	15730	±	321.3	848.6	±	51.19	21100	±	575.3	18150	±	476.7
728.6	PC_32:3_	13.02	±	5.544	180.6	±	34.76	111.5	±	8.632	7.420	±	0.5756	178.1	±	6.801	145.2	±	6.336
762.6	PC_34:0_	58.41	±	30.72	871.9	±	185.5	568.0	±	68.34	20.17	±	1.551	1330	±	49.91	1021	±	23.05
760.6	PC_34:1_	732.1	±	400.1	9098	±	1585	6582	±	630.5	242.0	±	19.40	12280	±	310.5	10350	±	255.4
758.6	PC_34:2_	2160	±	950.7	21750	±	3104	15970	±	1019	994.2	±	79.74	26480	±	1039	22630	±	677.1
756.6	PC_34:3_	30.96	±	11.83	723.8	±	99.53	471.8	±	40.84	9.200	±	0.7072	701.7	±	41.35	511.0	±	21.38
790.6	PC_36:0_/PC_O-38:7_	66.04	±	32.51	1184	±	273.0	724.5	±	78.85	26.51	±	1.895	1713	±	101.0	1284	±	19.39
788.6	PC_36:1_	229.1	±	128.6	3757	±	894.3	2473	±	328.0	88.30	±	7.673	6363	±	250.1	5089	±	91.89
786.6	PC_36:2_	363.0	±	208.4	7887	±	1739	5225	±	700.1	191.4	±	24.77	12570	±	457.6	10480	±	284.8
784.6	PC_36:3_	13.04	±	4.153	307.4	±	49.81	198.6	±	15.37	10.10	±	3.238	426.2	±	31.01	272.9	±	17.02
818.7	PC_38:0_/PC_O-40:7_	30.93	±	16.56	719.9	±	207.9	406.1	±	63.08	12.25	±	0.9141	1238	±	68.09	912.3	±	21.49
816.7	PC_38:1_	31.75	±	17.62	600.7	±	155.7	414.6	±	63.58	11.16	±	1.722	1025	±	124.4	642.0	±	20.49
814.7	PC_38:2_	12.70	±	4.775	186.2	±	54.68	110.4	±	13.51	8.704	±	1.427	427.8	±	22.23	327.2	±	4.862
812.7	PC_38:3_	1.828	±	0.5803	25.18	±	7.371	16.47	±	1.872	3.379	±	2.612	26.73	±	1.514	25.68	±	6.485
804.7	PC_38:7_/PC_O-38:0_	8.385	±	2.191	68.10	±	17.49	41.89	±	4.876	5.183	±	0.8508	110.1	±	4.183	88.95	±	3.079
802.7	PC_38:8_/PC_O-38:1_	6.127	±	1.308	35.02	±	7.180	28.57	±	3.597	4.617	±	0.5366	62.29	±	2.935	47.75	±	1.806
846.7	PC_40:0_/PC_O-42:7_	6.896	±	3.976	144.8	±	46.79	79.55	±	13.99	3.496	±	0.2167	317.7	±	13.37	225.8	±	8.343
884.7	PC_44:9_/PC_O-44:2_	5.582	±	0.5106	8.132	±	1.256	11.12	±	0.3921	3.969	±	0.8839	14.93	±	0.9244	15.26	±	0.3037
664.5	PC_O-28:0_	7.518	±	1.585	17.87	±	1.539	16.70	±	1.438	7.972	±	0.9606	19.74	±	1.069	16.81	±	1.100
662.5	PC_O-28:1_	23.98	±	0.901	30.39	±	1.659	28.94	±	1.987	21.04	±	0.7912	29.96	±	2.426	26.35	±	0.8645
660.5	PC_O-28:2_	137.3	±	5.370	117.3	±	3.043	128.7	±	9.205	122.8	±	3.659	118.2	±	6.551	115.8	±	4.706
692.5	PC_O-30:0_	21.32	±	4.084	30.14	±	1.279	37.36	±	5.624	16.76	±	1.846	31.61	±	3.485	25.08	±	2.243
690.5	PC_O-30:1_	58.96	±	10.22	59.81	±	3.559	100.2	±	24.34	65.90	±	26.09	60.82	±	9.116	48.96	±	2.396
688.5	PC_O-30:2_	8.027	±	1.337	21.63	±	1.846	16.85	±	1.230	8.266	±	1.138	15.65	±	0.7216	13.60	±	1.157
720.6	PC_O-32:0_	16.82	±	3.332	107.2	±	14.44	77.49	±	7.725	12.65	±	1.864	96.37	±	5.962	92.03	±	3.951
718.6	PC_O-32:1_	84.65	±	10.34	333.6	±	28.25	253.1	±	23.64	60.11	±	2.245	262.3	±	12.05	221.0	±	8.740
716.6	PC_O-32:2_	27.60	±	9.088	446.0	±	60.11	281.5	±	21.65	9.555	±	0.9663	304.0	±	17.95	251.8	±	2.698

748.6	PC_O-34:0_	20.91	±	7.709	287.4	±	67.47	149.8	±	20.72	11.90	±	1.278	302.1	±	21.96	245.1	±	9.511
746.6	PC_O-34:1_	24.82	±	8.322	310.7	±	55.38	219.7	±	24.07	12.51	±	1.384	333.7	±	14.36	260.3	±	6.275
744.6	PC_O-34:2_	25.25	±	12.45	538.0	±	86.46	334.9	±	24.60	9.960	±	0.8987	577.5	±	28.06	431.6	±	9.686
776.6	PC_O-36:0_	23.33	±	8.930	178.0	±	49.74	98.28	±	12.58	13.88	±	0.7755	259.8	±	10.26	219.2	±	5.974
774.6	PC_O-36:1_	21.13	±	5.894	160.0	±	32.12	133.4	±	19.62	11.95	±	1.305	264.6	±	21.50	195.7	±	4.378
772.6	PC_O-36:2_	8.850	±	3.497	184.4	±	34.89	113.1	±	11.16	5.309	±	0.6325	272.0	±	13.92	208.4	±	4.244
800.7	PC_O-38:2_	4.218	±	0.4481	23.09	±	4.098	14.29	±	0.8367	3.764	±	0.3134	29.76	±	0.5652	23.38	±	0.9401
454.3	LPE_16:0_	366.4	±	41.49	362.7	±	91.40	775.6	±	138.4	169.9	±	28.02	618.4	±	24.72	1220	±	69.38
452.3	LPE_16:1_	1046	±	148.7	2947	±	828.9	6459	±	1418	983.4	±	200.0	7922	±	508.1	12640	±	646.6
482.3	LPE_18:0_	78.90	±	14.06	200.3	±	62.66	245.2	±	63.24	89.03	±	16.15	684.8	±	50.48	419.1	±	32.18
480.3	LPE_18:1_	2997	±	365.4	8738	±	2666	10050	±	2800	3276	±	581.9	29670	±	1665	18560	±	552.7
478.3	LPE_18:2_	B.Q.L.	±	B.Q.L.	52.74	±	13.24	44.03	±	7.682	10.05	±	4.628	69.20	±	7.117	37.98	±	4.063
608.5	PE_26:0_	3.462	±	1.680	34.59	±	5.043	23.69	±	1.446	1.064	±	0.6743	22.35	±	1.285	20.37	±	1.317
606.5	PE_26:1_	7.439	±	2.321	37.54	±	5.972	25.76	±	1.159	6.340	±	1.027	26.69	±	1.358	20.40	±	2.043
636.5	PE_28:0_	4.824	±	1.886	103.6	±	11.00	67.03	±	3.225	0.7979	±	0.7979	55.04	±	2.949	45.40	±	1.252
634.5	PE_28:1_	26.70	±	9.749	273.3	±	31.58	189.1	±	7.074	8.935	±	0.5977	221.1	±	8.888	152.5	±	2.325
664.5	PE_30:0_	3.571	±	1.340	49.52	±	5.125	34.85	±	1.777	1.346	±	0.8546	33.77	±	1.763	29.68	±	1.984
662.5	PE_30:1_	56.60	±	10.31	472.5	±	55.28	355.1	±	16.53	39.96	±	1.294	376.7	±	11.21	294.6	±	6.930
660.5	PE_30:2_	3.461	±	1.937	64.00	±	11.24	42.25	±	1.628	2.040	±	0.9412	57.13	±	2.991	40.49	±	2.418
692.5	PE_32:0_	22.81	±	4.522	306.3	±	34.63	224.3	±	12.34	18.16	±	1.730	254.8	±	12.06	193.1	±	7.762
690.5	PE_32:1_	1522	±	135.9	6064	±	557.0	4812	±	221.7	1376	±	28.61	5186	±	156.4	4262	±	120.2
686.5	PE_32:3_	5.419	±	1.726	14.54	±	1.848	12.38	±	1.183	4.535	±	0.9771	15.96	±	0.9864	12.60	±	0.8093
720.6	PE_34:0_	27.22	±	9.038	440.2	±	70.05	340.9	±	22.26	13.20	±	2.589	521.5	±	16.05	393.7	±	19.05
718.6	PE_34:1_	495.4	±	166.3	8399	±	1300	6508	±	405.5	227.7	±	47.65	10290	±	202.5	7972	±	263.2
716.6	PE_34:2_	2233	±	838.6	33560	±	4406	25050	±	1686	1148	±	218.1	40820	±	537.8	34140	±	606.3
714.6	PE_34:3_	9.530	±	2.961	183.1	±	11.37	137.1	±	18.21	3.282	±	1.144	136.7	±	11.09	100.8	±	7.134
712.6	PE_34:4_	49.14	±	9.735	115.4	±	8.652	115.4	±	16.49	35.06	±	5.439	95.58	±	10.31	81.79	±	6.185
748.6	PE_36:0	0.6437	±	0.6437	14.68	±	3.254	12.70	±	1.233	B.Q.L.	±	B.Q.L.	32.75	±	1.661	26.66	±	1.454
746.6	PE_36:1_	30.40	±	8.108	636.7	±	122.8	470.4	±	47.74	19.35	±	3.415	1189	±	19.60	918.1	±	21.84
744.6	PE_36:2_	268.8	±	76.80	7455	±	1421	5026	±	528.7	174.1	±	40.55	12640	±	337.0	9731	±	85.09
742.6	PE_36:3_	5.119	±	1.859	176.4	±	9.444	125.9	±	17.76	3.041	±	0.9974	127.9	±	7.411	95.73	±	5.902
740.6	PE_36:4_	16.59	±	7.172	191.9	±	25.96	173.8	±	22.42	6.701	±	1.404	229.1	±	21.52	152.5	±	11.29
772.6	PE_38:2_	B.Q.L.	±	B.Q.L.	12.18	±	2.519	8.735	±	1.314	B.Q.L.	±	B.Q.L.	21.72	±	2.373	19.52	±	1.211
768.6	PE_38:4_	B.Q.L.	±	B.Q.L.	15.54	±	3.538	16.96	±	2.790	3.034	±	2.330	26.02	±	1.817	21.51	±	1.773
722.6	PE_O-36:6_	11.23	±	4.297	423.0	±	53.48	168.7	±	66.82	9.839	±	2.527	415.1	±	49.27	425.3	±	16.29

Supplementary Table 2: Concentrations (in pmol/mg protein) of cholesteryl esters (CE), diacylglycerols (DAG), and triacylglycerols (TAG) in Aac2 and ArANT expressing yeast cells, Percoll-purified mitochondria and mitoplasts. For TAG determination, all the fragments that would comprise the acyl chains were summed up and isomeric composition for the molecular species as the brutto nomenclature (carbon:double bond) is given. CE concentrations are compared to the internal standard TAG_17:1-17:1-17:1. N.D.: not determined. B.Q.L.: below quantitative limit. Results shown are Mean +/- S.E.M.

		Aac2			Artemia-ANT		
**$[M+NH4]^+$**	**Species**	**Yeast Cell**	**Mitochondria**	**Mitoplasts**	**Yeast Cell**	**Mitochondria**	**Mitoplasts**
666.5	CE_18:2	12.53 ± 3.105	19.08 ± 8.751	23.73 ± 9.218	18.73 ± 7.045	7.161 ± 3.305	15.48 ± 9.161
668.5	CE_18:1	3.609 ± 1.616	7.378 ± 2.406	4.626 ± 2.166	5.496 ± 1.913	4.437 ± 1.544	5.567 ± 2.867
528.4	DAG_28:1	2.556 ± 1.617	32.67 ± 6.126	14.63 ± 0.7781	3.752 ± 2.562	27.14 ± 2.883	14.91 ± 1.447
530.4	DAG_28:0	88.57 ± 7.099	59.69 ± 5.554	67.29 ± 5.256	87.12 ± 5.540	53.00 ± 3.031	59.27 ± 6.821
554.4	DAG_30:2	10.33 ± 4.945	206.8 ± 45.00	86.33 ± 5.924	15.74 ± 2.879	239.8 ± 15.84	92.68 ± 3.098
556.4	DAG_30:1	54.85 ± 15.55	1193 ± 216.3	587.2 ± 54.25	57.35 ± 14.19	1365 ± 62.20	751.6 ± 28.21
558.4	DAG_30:0	283.7 ± 11.97	351.3 ± 17.17	342.2 ± 12.38	267.3 ± 5.037	415.5 ± 12.11	367.4 ± 16.41
582.4	DAG_32:2	280.1 ± 129.1	7650 ± 1724	3108 ± 153.1	143.5 ± 18.28	10820 ± 762.8	4487 ± 176.8
584.4	DAG_32:1	333.0 ± 152.8	11610 ± 2438	6005 ± 791.0	215.5 ± 35.76	16180 ± 738.5	10260 ± 400.0
586.4	DAG_32:0	1498 ± 52.06	1683 ± 50.18	1608 ± 46.92	1465 ± 44.71	1786 ± 61.98	1771 ± 68.42
608.5	DAG_34:3	23.07 ± 3.591	188.3 ± 33.83	103.8 ± 3.054	26.12 ± 2.190	209.3 ± 14.05	130.0 ± 1.904
610.5	DAG_34:2	633.4 ± 328.7	16210 ± 4031	7144 ± 667.7	356.3 ± 37.92	30830 ± 1825	14910 ± 240.1
612.5	DAG_34:1	589.6 ± 313.6	23170 ± 5770	12330 ± 2095	315.6 ± 23.89	41280 ± 2346	28380 ± 699.7
614.5	DAG_34:0	4756 ± 148.5	5361 ± 163.0	5054 ± 98.80	4478 ± 141.6	5733 ± 180.4	5508 ± 127.5
636.5	DAG_36:3	34.08 ± 4.854	135.1 ± 30.15	79.13 ± 6.695	45.70 ± 8.862	149.7 ± 9.570	94.97 ± 5.108
638.5	DAG_36:2	190.5 ± 81.02	3111 ± 881.4	2246 ± 318.7	143.2 ± 13.22	8598 ± 522.0	6862 ± 189.9
640.5	DAG_36:1	254.1 ± 102.3	6368 ± 1793	3755 ± 666.4	183.7 ± 12.07	14250 ± 1056	10700 ± 256.8
642.5	DAG_36:0	4243 ± 133.6	4362 ± 122.9	4340 ± 84.43	4089 ± 98.08	4734 ± 120.7	4554 ± 114.2
684.5	TAG_38:0	15.54 ± 1.606	96.68 ± 71.98	18.93 ± 1.055	12.73 ± 0.3978	18.86 ± 1.795	18.72 ± 1.104
710.6	TAG_40:1	7.374 ± 3.477	100.3 ± 44.42	20.27 ± 5.660	2.912 ± 1.334	35.06 ± 5.013	35.63 ± 1.728
712.6	TAG_40:0	478.5 ± 68.18	340.2 ± 54.64	441.4 ± 80.47	296.6 ± 49.06	255.9 ± 17.15	262.8 ± 9.239
736.6	TAG_42:2	42.70 ± 13.84	925.4 ± 164.3	276.9 ± 96.19	24.85 ± 2.364	593.5 ± 88.28	554.8 ± 12.18
738.6	TAG_42:1	51.16 ± 24.67	306.5 ± 88.05	94.78 ± 21.01	21.05 ± 1.230	192.6 ± 22.12	178.0 ± 7.259
740.6	TAG_42:0	16.27 ± 3.798	88.78 ± 65.29	18.76 ± 2.601	13.60 ± 2.190	21.30 ± 2.832	17.87 ± 0.9514
762.6	TAG_44:3	6.848 ± 2.200	11.36 ± 0.8695	4.878 ± 2.253	9.264 ± 0.675	8.202 ± 0.7587	9.346 ± 1.263
764.6	TAG_44:2	93.52 ± 16.08	60.75 ± 18.24	49.37 ± 9.637	40.42 ± 4.310	30.55 ± 2.598	41.87 ± 3.707
766.6	TAG_44:1	42.60 ± 20.32	51.92 ± 25.07	26.06 ± 2.189	22.92 ± 5.599	25.29 ± 7.435	31.93 ± 0.6687
768.6	TAG_44:0	161.0 ± 35.28	93.35 ± 27.28	70.75 ± 14.30	77.33 ± 8.541	50.88 ± 11.22	50.98 ± 12.45
790.6	TAG_46:3	9.429 ± 2.268	23.38 ± 7.712	7.117 ± 0.3515	6.968 ± 1.081	9.412 ± 2.022	8.512 ± 0.5838
792.6	TAG_46:2	29.12 ± 9.738	51.98 ± 16.73	21.27 ± 1.090	25.74 ± 5.964	29.02 ± 9.940	26.72 ± 1.459
794.6	TAG_46:1	41.86 ± 16.62	71.36 ± 35.98	35.24 ± 3.953	46.00 ± 14.76	47.20 ± 20.73	49.67 ± 2.060

796.6	TAG_46:0	37.54	±	3.193	56.13	±	15.76	42.65	±	3.818	56.16	±	15.16	53.13	±	19.18	32.87	±	1.641
812.7	TAG_48:6	1.818	±	1.169	9.320	±	1.040	5.938	±	1.291	3.725	±	1.804	8.228	±	0.7363	7.894	±	0.2991
818.7	TAG_48:3	120.7	±	62.22	228.9	±	44.15	46.63	±	3.832	45.57	±	2.595	79.61	±	10.04	86.21	±	5.426
820.7	TAG_48:2	122.5	±	57.32	143.1	±	28.52	71.85	±	4.809	60.97	±	11.74	96.18	±	19.68	137.4	±	3.639
822.7	TAG_48:1	43.23	±	11.49	79.26	±	32.85	41.70	±	4.206	55.33	±	18.20	64.48	±	24.76	49.23	±	2.102
824.7	TAG_48:0	110.3	±	9.548	112.8	±	10.97	117.7	±	7.025	121.7	±	16.10	116.0	±	20.90	96.00	±	4.657
842.7	TAG_50:5	5.547	±	0.3466	9.543	±	1.148	5.545	±	0.2850	4.000	±	0.8384	6.673	±	0.7226	6.686	±	0.5762
844.7	TAG_50:4	10.96	±	1.559	25.22	±	4.205	14.05	±	0.7409	9.111	±	0.9365	18.25	±	2.049	15.78	±	0.5396
846.7	TAG_50:3	344.9	±	191.8	518.5	±	98.18	120.8	±	4.919	129.8	±	7.452	219.1	±	17.89	359.9	±	21.69
848.7	TAG_50:2	318.5	±	176.9	304.3	±	67.53	193.9	±	18.67	126.5	±	14.20	227.0	±	25.63	603.7	±	18.47
850.7	TAG_50:1	85.33	±	24.16	141.4	±	57.31	77.21	±	9.359	75.97	±	13.09	101.0	±	21.58	118.0	±	6.205
852.7	TAG_50:0	210.4	±	20.04	195.3	±	6.600	216.0	±	9.800	195.2	±	14.58	192.4	±	13.45	175.0	±	14.21
870.7	TAG_52:5	7.456	±	0.4955	16.89	±	5.230	6.733	±	1.604	5.096	±	1.111	9.816	±	1.057	9.971	±	0.6115
872.7	TAG_52:4	19.87	±	2.365	52.83	±	21.47	22.00	±	1.656	20.64	±	2.308	26.21	±	2.815	26.43	±	2.663
874.7	TAG_52:3	311.5	±	163.4	399.3	±	89.96	145.9	±	14.27	159.2	±	12.06	233.2	±	19.21	573.9	±	26.06
876.7	TAG_52:2	309.3	±	160.9	305.0	±	104.0	234.4	±	30.12	137.3	±	12.05	235.7	±	26.27	913.3	±	16.53
878.7	TAG_52:1	60.04	±	20.69	96.88	±	39.33	54.87	±	5.372	44.16	±	4.904	62.75	±	8.030	114.9	±	2.671
880.7	TAG_52:0	233.9	±	27.90	224.9	±	11.52	234.4	±	10.81	215.8	±	22.35	211.4	±	13.88	203.7	±	19.64
896.7	TAG_54:6	28.48	±	3.693	73.18	±	31.36	35.59	±	3.089	27.54	±	2.589	38.13	±	4.911	41.00	±	2.350
898.7	TAG_54:5	25.98	±	4.057	101.2	±	65.86	27.98	±	3.452	27.25	±	3.734	27.75	±	3.632	30.91	±	3.756
900.8	TAG_54:4	40.84	±	6.007	160.4	±	103.5	39.54	±	5.335	41.60	±	5.851	40.10	±	5.204	44.69	±	5.427
902.8	TAG_54:3	103.4	±	26.26	286.1	±	164.1	79.83	±	10.70	81.16	±	9.947	98.24	±	10.86	210.7	±	15.44
904.8	TAG_54:2	77.53	±	31.68	122.5	±	48.37	69.20	±	8.736	46.68	±	4.393	72.02	±	4.937	246.5	±	8.624
906.8	TAG_54:1	22.13	±	5.303	38.78	±	12.40	19.91	±	2.013	17.99	±	1.915	23.97	±	2.517	37.01	±	1.305
908.8	TAG_54:0	118.4	±	14.67	112.5	±	7.088	124.9	±	7.992	107.2	±	13.66	110.5	±	8.322	106.0	±	9.372
924.8	TAG_56:6	24.33	±	2.730	23.28	±	1.321	26.69	±	1.953	21.81	±	2.447	26.33	±	2.495	29.72	±	2.671
928.8	TAG_56:4	3.554	±	1.134	14.79	±	4.806	7.614	±	0.6003	5.306	±	1.235	9.891	±	0.6146	10.57	±	0.5166
930.8	TAG_56:3	6.937	±	0.6418	24.49	±	9.215	10.29	±	0.8970	7.279	±	0.4653	15.82	±	1.091	14.52	±	0.6809
932.8	TAG_56:2	11.73	±	1.104	25.90	±	8.085	13.74	±	1.055	11.92	±	1.005	19.97	±	1.387	19.04	±	0.4274
934.8	TAG_56:1	10.93	±	1.330	18.16	±	5.855	10.59	±	1.451	14.49	±	0.9455	13.32	±	2.194	10.80	±	0.4633
936.8	TAG_56:0	6.431	±	0.6806	6.763	±	0.8326	6.865	±	0.1861	7.188	±	1.479	7.212	±	1.405	5.544	±	0.3396
954.8	TAG_58:5	5.767	±	1.244	7.864	±	0.4943	8.561	±	0.8993	6.234	±	0.2124	7.575	±	0.6691	8.765	±	0.4720
956.8	TAG_58:4	18.37	±	2.156	17.47	±	1.141	22.04	±	1.940	18.28	±	1.766	18.09	±	1.297	19.06	±	0.7642
958.8	TAG_58:3	268.8	±	26.55	226.9	±	26.81	319.1	±	31.42	241.5	±	30.19	261.5	±	18.47	273.0	±	18.29
960.8	TAG_58:2	15.80	±	1.684	22.51	±	4.225	18.02	±	1.578	16.49	±	1.369	18.19	±	1.559	19.09	±	1.222
962.8	TAG_58:1	8.197	±	1.632	9.493	±	2.559	6.664	±	0.6994	8.475	±	0.4237	7.411	±	1.411	5.597	±	0.5167

Supplementary Table 3: Concentrations (in pmol/mg protein) of lysophosphatidic acids (LPA), phosphatidic acids (PA), lysophosphatidylglycerols (LPG), phosphatidylglycerols (PG), lysophosphatidylinositols (LPI), phosphatidylinositols (PI), lysophosphatidylserines (LPS), and phosphatidylserine (PS) in Aac2 and ArANT expressing yeast cells, Percoll-purified mitochondria and mitoplasts. N.D.: not determined. B.Q.L.: below quantitative limit. p(carbond:double bond) indicates a plasmalogen species. . a(carbon:double bond) indicates an alkylenyl species. Results shown are Mean +/- S.E.M.

		Aac2									Artemia-ANT								
$[M-H]^-$	Species	Yeast Cell			Mitochondria			Mitoplasts			Yeast Cell			Mitochondria			Mitoplasts		
409.3	LPA_16:0_	683.1	±	121.2	180.2	±	17.90	380.6	±	115.1	435.9	±	125.4	134.5	±	21.98	128.3	±	10.02
407.3	LPA_16:1_	558.1	±	131.0	113.7	±	12.80	272.1	±	57.61	238.1	±	82.06	154.3	±	23.23	171.5	±	24.03
437.3	LPA_18:0_	632.8	±	118.5	132.3	±	25.59	319.0	±	76.28	368.8	±	108.9	99.55	±	20.71	99.40	±	14.72
435.3	LPA_18:1_	235.8	±	52.76	69.37	±	9.324	130.5	±	31.40	141.6	±	46.04	116.4	±	15.92	106.7	±	4.006
535.4	PA_10:0/14:0	190.0	±	21.18	106.1	±	22.62	140.8	±	30.04	119.2	±	18.47	55.99	±	9.277	68.60	±	6.949
563.4	PA_12:0/14:0	59.76	±	11.85	36.88	±	6.713	46.95	±	12.52	51.77	±	8.620	14.33	±	4.715	30.31	±	6.388
591.4	PA_14:0/14:0	166.0	±	21.27	105.0	±	18.80	114.6	±	11.41	107.0	±	14.03	72.98	±	6.600	84.05	±	11.07
617.5	PA_14:0/16:1	345.0	±	58.76	269.9	±	23.31	336.6	±	12.59	254.2	±	29.19	257.6	±	29.62	267.2	±	32.86
647.5	PA_14:0/18:0	533.3	±	73.24	223.9	±	39.53	336.7	±	58.00	322.6	±	19.10	135.9	±	20.03	161.6	±	19.48
645.5	PA_14:0/18:1	298.6	±	34.78	1013	±	114.2	701.9	±	35.88	233.4	±	26.59	570.1	±	40.41	629.1	±	64.26
643.5	PA_14:0/18:2	69.01	±	11.60	56.82	±	11.21	59.46	±	10.48	60.52	±	8.275	33.43	±	4.817	32.07	±	4.685
641.5	PA_14:0/18:3	54.13	±	12.43	59.41	±	18.39	47.52	±	6.563	65.55	±	15.35	53.67	±	11.44	39.77	±	5.303
675.5	PA_14:0/20:0	79.35	±	10.03	112.3	±	19.53	73.98	±	10.28	57.05	±	6.290	35.17	±	4.607	29.25	±	2.419
673.5	PA_14:0/20:1	70.05	±	9.039	178.7	±	25.25	137.7	±	21.60	64.69	±	12.49	97.58	±	8.796	101.2	±	7.812
671.5	PA_14:0/20:2	49.32	±	7.497	54.23	±	7.928	41.48	±	6.464	35.21	±	6.258	33.81	±	4.376	48.20	±	3.425
669.5	PA_14:0/20:3	34.02	±	8.216	15.34	±	5.489	32.85	±	9.952	16.17	±	4.274	8.123	±	3.850	15.51	±	6.161
667.5	PA_14:0/20:4	94.97	±	23.14	38.28	±	7.409	62.91	±	15.47	62.62	±	7.825	34.65	±	12.43	46.89	±	3.916
703.6	PA_14:0/22:0	108.9	±	19.32	148.5	±	22.27	103.3	±	12.73	87.2	±	8.853	62.92	±	6.656	64.35	±	4.967
701.6	PA_14:0/22:1	56.65	±	10.85	67.70	±	7.271	66.01	±	3.730	36.49	±	7.485	54.17	±	3.829	51.31	±	7.625
647.5	PA_16:0/16:0	543.5	±	46.35	733.8	±	97.36	740.6	±	64.51	424.0	±	34.43	554.8	±	75.83	526.3	±	57.09
645.5	PA_16:0/16:1	852.7	±	84.99	1917	±	151.7	1479	±	68.88	620.6	±	36.87	1274	±	94.89	1236	±	126.9
675.5	PA_16:0/18:0	529.6	±	50.25	491.1	±	68.55	557.0	±	94.04	450.5	±	36.97	332.0	±	32.41	345.7	±	50.63
673.5	PA_16:0/18:1	549.4	±	61.81	1391	±	132.1	1115	±	72.43	422.9	±	39.10	1174	±	126.7	1043	±	94.80
671.5	PA_16:0/18:2	293.7	±	28.65	222.3	±	25.20	318.1	±	57.65	243.0	±	26.48	186.0	±	14.89	177.2	±	12.69
669.5	PA_16:0/18:3	483.8	±	112.9	424.1	±	138.6	328.9	±	39.67	628.8	±	196.1	324.6	±	43.05	304.6	±	40.60
703.6	PA_16:0/20:0	525.7	±	51.96	898.1	±	127.6	511.3	±	57.77	451.3	±	41.27	302.4	±	26.50	277.7	±	24.78
701.6	PA_16:0/20:1	772.4	±	41.23	2056	±	172.3	1368	±	102.7	620.1	±	38.04	1424	±	83.53	1322	±	102.4
699.5	PA_16:0/20:2	496.4	±	26.64	565.2	±	54.23	488.6	±	45.60	431.9	±	37.78	457.7	±	48.08	439.6	±	37.23
697.5	PA_16:0/20:3	273.2	±	24.54	146.7	±	13.81	220.8	±	18.12	187.8	±	16.00	154.1	±	21.05	169.6	±	21.11
695.5	PA_16:0/20:4	318.3	±	37.45	175.1	±	20.04	308.7	±	68.51	211.2	±	31.03	164.2	±	26.53	160.5	±	15.42

693.5	PA_16:0/20:5	196.0	±	19.84	158.0	±	15.69	289.1	±	85.28	204.1	±	22.36	115.3	±	16.00	125.2	±	8.953
731.6	PA_16:0/22:0	602.5	±	56.34	1531	±	135.8	726.3	±	46.66	495.7	±	45.33	585.5	±	76.02	509.5	±	67.46
729.6	PA_16:0/22:1	172.3	±	25.34	141.1	±	20.10	158.0	±	16.00	154.0	±	14.61	141.5	±	20.42	156.8	±	9.040
727.6	PA_16:0/22:2	237.2	±	29.78	152.4	±	20.25	214.7	±	20.52	170.9	±	22.07	195.8	±	12.80	187.0	±	24.13
725.6	PA_16:0/22:3	429.2	±	72.06	447.5	±	37.20	473.7	±	123.1	207.4	±	16.11	225.1	±	24.31	228.2	±	33.68
723.6	PA_16:0/22:4	274.2	±	31.55	142.5	±	19.88	241.3	±	60.26	192.7	±	13.89	111.0	±	19.39	124.9	±	18.63
721.6	PA_16:0/22:5	216.1	±	23.27	114.6	±	9.612	193.1	±	53.27	145.7	±	9.882	114.3	±	10.06	113.6	±	9.804
719.6	PA_16:0/22:6	543.7	±	47.96	610.0	±	46.28	644.9	±	66.73	377.3	±	35.96	507.6	±	38.68	530.8	±	52.47
703.6	PA_18:0/18:0	594.2	±	44.89	309.1	±	35.88	491.5	±	124.8	455.9	±	42.06	213.1	±	26.23	223.2	±	25.02
701.6	PA_18:0/18:1	798.0	±	89.36	3591	±	444.1	2377	±	159.3	625.3	±	73.44	2678	±	180.4	2467	±	203.4
699.5	PA_18:0/18:2	294.4	±	33.75	675.9	±	112.7	340.1	±	74.20	248.3	±	24.20	189.3	±	20.86	209.3	±	16.84
699.5	PA_18:1/18:1	1260	±	220.9	10620	±	1334	6639	±	315.9	967.6	±	180.4	10140	±	691.6	8996	±	795.4
697.5	PA_18:0/18:3	467.0	±	111.2	416.2	±	168.7	272.9	±	39.93	682.7	±	220.4	226.4	±	35.14	261.4	±	30.17
731.6	PA_18:0/20:0	577.7	±	47.02	421.6	±	73.15	393.2	±	39.86	512.5	±	32.34	204.2	±	13.74	188.9	±	19.92
729.6	PA_18:0/20:1	612.2	±	40.23	429.0	±	40.75	472.6	±	54.26	510.8	±	70.10	362.7	±	15.93	420.6	±	50.59
727.6	PA_18:0/20:2	657.0	±	61.73	340.3	±	43.44	421.5	±	35.51	467.9	±	59.80	256.2	±	15.05	314.8	±	27.39
725.6	PA_18:0/20:3	287.6	±	34.39	120.5	±	17.49	265.7	±	36.54	200.8	±	16.53	103.2	±	11.63	131.5	±	17.79
723.6	PA_18:0/20:4	291.1	±	31.24	107.0	±	13.86	236.1	±	47.98	206.5	±	26.05	77.29	±	8.976	114.5	±	16.80
721.6	PA_18:0/20:5	188.3	±	15.78	108.6	±	25.48	214.9	±	61.51	189.7	±	19.24	70.84	±	11.86	94.08	±	8.405
759.6	PA_18:0/22:0	503.9	±	76.88	566.9	±	58.12	404.2	±	32.43	454.6	±	42.25	260.2	±	15.35	314.4	±	35.09
757.6	PA_18:0/22:1	181.3	±	20.40	102.8	±	12.45	124.4	±	12.73	137.2	±	11.48	98.98	±	10.45	114.5	±	13.44
755.6	PA_18:0/22:2	235.2	±	29.34	98.07	±	16.50	136.7	±	12.33	169.3	±	13.98	99.52	±	12.85	128.1	±	15.49
753.6	PA_18:0/22:3	268.2	±	32.68	195.8	±	30.19	259.4	±	61.22	155.7	±	17.04	127.9	±	19.39	131.5	±	15.67
751.6	PA_18:0/22:4	245.1	±	31.60	109.5	±	20.06	170.9	±	15.98	165.5	±	15.49	58.23	±	5.975	70.12	±	12.23
749.6	PA_18:0/22:5	190.9	±	23.83	121.2	±	16.59	172.0	±	29.58	152.1	±	17.46	107.6	±	9.423	106.5	±	9.866
747.6	PA_18:0/22:6	513.0	±	42.54	291.7	±	27.63	357.1	±	36.66	437.0	±	39.89	224.8	±	25.49	244.6	±	31.25
695.5	PA_18:1/18:3	180.2	±	35.97	224.2	±	32.71	180.4	±	11.07	318.8	±	104.5	365.4	±	56.30	369.0	±	61.14
725.6	PA_18:1/20:2	318.5	±	44.37	555.4	±	55.61	483.8	±	40.99	276.8	±	35.21	573.3	±	44.80	545.3	±	46.07
721.6	PA_18:1/20:4	170.5	±	26.50	130.2	±	18.14	194.9	±	18.79	106.4	±	9.151	234.0	±	15.38	258.1	±	21.14
757.6	PA_18:1/22:0	231.6	±	49.68	2127	±	381.6	1068	±	208.4	197.9	±	16.02	1819	±	132.4	1785	±	205.1
659.5	PA_p16:0/18:0	1179	±	84.57	595.6	±	68.76	667.7	±	61.45	872.2	±	76.03	307.5	±	24.02	396.2	±	32.39
657.5	PA_p16:0/18:1	7.989	±	4.090	31.47	±	6.618	40.40	±	6.078	3.758	±	2.394	17.13	±	4.686	16.86	±	4.192
631.5	PA_p18:0/14:0	71.59	±	12.39	101.7	±	13.21	87.87	±	19.03	50.10	±	7.280	57.83	±	4.633	61.92	±	8.465
659.5	PA_p18:0/16:0	504.0	±	39.56	738.0	±	51.42	739.9	±	152.3	414.4	±	38.19	442.2	±	37.72	449.9	±	56.33
687.5	PA_p18:0/18:0	498.0	±	35.41	304.3	±	32.13	489.8	±	125.5	386.2	±	30.81	217.8	±	25.31	201.6	±	17.66
685.5	PA_p18:0/18:1	481.0	±	58.47	3274	±	441.2	2247	±	139.3	363.7	±	48.68	2683	±	179.8	2345	±	232.7

455.3	LPG_14:0_	18.61	±	3.261	11.69	±	2.016	10.62	±	1.408	27.53	±	4.652	11.60	±	0.5764	9.116	±	1.145
483.3	LPG_16:0_	298.5	±	56.03	176.8	±	20.96	162.6	±	22.56	447.5	±	68.45	149.7	±	6.871	139.7	±	8.104
481.3	LPG_16:1_	219.9	±	53.43	139.7	±	28.23	138.9	±	30.97	307.6	±	72.59	252.5	±	35.04	181.6	±	4.255
511.4	LPG_18:0_	360.2	±	58.95	189.7	±	20.41	178.0	±	30.54	538.0	±	88.91	138.9	±	5.851	131.5	±	6.477
509.4	LPG_18:1_	125.0	±	24.79	100.2	±	17.15	125.9	±	32.05	224.9	±	38.34	219.4	±	23.91	192.7	±	9.758
539.4	LPG_20:0_	8.051	±	1.447	5.902	±	0.759	3.916	±	1.774	12.24	±	2.437	1.552	±	1.000	3.165	±	1.038
665.5	PG_14:0/14:0	18.87	±	0.3691	16.04	±	0.8301	18.42	±	2.225	21.60	±	1.681	13.55	±	1.044	15.48	±	1.271
693.5	PG_14:0/16:0	21.02	±	3.339	17.76	±	1.374	25.67	±	9.939	39.75	±	6.276	15.57	±	1.532	15.73	±	1.629
691.5	PG_14:0/16:1	44.08	±	9.178	38.75	±	3.183	35.85	±	5.515	37.67	±	4.627	35.87	±	1.818	34.77	±	1.753
721.6	PG_14:0/18:0	21.38	±	3.291	12.12	±	1.603	20.13	±	7.005	35.88	±	5.172	11.04	±	0.895	11.95	±	0.9244
719.6	PG_14:0/18:1	16.35	±	2.275	46.68	±	8.546	35.64	±	6.091	18.05	±	2.951	59.97	±	1.232	45.80	±	2.452
717.6	PG_14:0/18:2	2.188	±	1.436	6.238	±	1.415	6.650	±	1.802	3.767	±	0.8059	6.129	±	1.466	6.195	±	0.5748
749.6	PG_14:0/20:0	123.8	±	49.62	62.85	±	14.56	49.56	±	18.52	202.6	±	51.32	63.6	±	20.27	63.63	±	16.82
747.6	PG_14:0/20:1	23.77	±	2.320	17.18	±	1.807	16.19	±	1.227	26.57	±	3.354	16.28	±	1.265	18.98	±	0.7324
741.6	PG_14:0/20:4	3.294	±	1.473	7.964	±	0.8921	5.393	±	2.088	4.626	±	1.822	5.904	±	1.377	6.922	±	0.8188
739.6	PG_14:0/20:5	7.548	±	0.8526	18.74	±	1.295	13.29	±	2.894	7.613	±	1.308	15.43	±	2.418	15.19	±	0.7678
777.6	PG_14:0/22:0	6.132	±	2.338	4.847	±	0.3599	4.639	±	1.848	7.707	±	1.137	3.348	±	1.075	3.929	±	0.8297
767.6	PG_14:0/22:5	4.620	±	2.027	7.627	±	0.6825	5.343	±	1.33	8.791	±	1.754	8.253	±	0.9644	7.514	±	1.084
721.6	PG_16:0/16:0	35.82	±	5.167	21.59	±	0.9761	32.39	±	12.67	45.61	±	6.858	25.46	±	1.889	22.57	±	1.206
719.6	PG_16:0/16:1	67.31	±	10.81	146.5	±	17.10	105.9	±	7.481	82.10	±	13.13	152.7	±	4.786	124.2	±	2.625
749.6	PG_16:0/18:0	27.31	±	2.954	30.47	±	4.014	30.24	±	4.488	38.16	±	5.512	51.48	±	4.722	44.05	±	3.492
747.6	PG_16:0/18:1	40.33	±	5.899	315.1	±	53.19	193.7	±	13.80	40.17	±	4.724	491.0	±	11.72	355.6	±	9.767
745.6	PG_16:0/18:2	16.46	±	3.204	43.48	±	5.802	33.58	±	6.352	24.83	±	2.320	51.38	±	4.768	46.98	±	5.480
743.6	PG_16:0/18:3	11.91	±	1.835	9.514	±	0.9768	11.38	±	2.001	13.33	±	0.9484	17.09	±	1.179	17.29	±	1.981
777.6	PG_16:0/20:0	31.91	±	5.222	24.56	±	1.587	24.83	±	3.512	41.16	±	4.674	26.56	±	1.561	25.68	±	1.579
775.6	PG_16:0/20:1	18.40	±	2.925	32.67	±	4.946	42.27	±	5.965	18.90	±	3.321	46.65	±	5.315	49.34	±	5.389
773.6	PG_16:0/20:2	8.91	±	1.431	17.42	±	3.966	20.91	±	5.016	8.743	±	1.265	27.83	±	6.195	29.31	±	5.360
771.6	PG_16:0/20:3	11.51	±	2.233	28.39	±	7.774	21.12	±	2.795	15.31	±	1.398	84.96	±	6.609	61.99	±	5.167
769.6	PG_16:0/20:4	10.07	±	1.270	25.48	±	4.956	20.03	±	4.098	14.25	±	2.004	35.15	±	4.266	29.65	±	3.166
767.6	PG_16:0/20:5	19.69	±	2.516	67.67	±	8.716	47.25	±	9.529	27.24	±	4.469	77.84	±	6.185	68.07	±	4.247
805.7	PG_16:0/22:0	59.25	±	10.63	38.33	±	3.916	41.05	±	5.707	84.25	±	10.69	37.41	±	2.391	39.43	±	3.484
803.7	PG_16:0/22:1	8.801	±	1.426	6.198	±	0.8082	5.925	±	0.656	10.30	±	1.919	6.445	±	1.381	6.041	±	0.6865
801.7	PG_16:0/22:2	12.04	±	1.841	7.792	±	0.5152	8.105	±	0.8135	19.27	±	2.544	7.419	±	0.4443	7.598	±	0.7094
799.6	PG_16:0/22:3	8.614	±	1.411	12.45	±	1.648	13.25	±	1.441	10.33	±	1.533	16.24	±	1.037	13.01	±	0.5811
797.6	PG_16:0/22:4	11.07	±	1.399	18.38	±	2.657	14.80	±	3.557	15.74	±	2.250	25.16	±	3.176	18.20	±	1.438
795.6	PG_16:0/22:5	52.23	±	10.36	48.54	±	3.012	42.23	±	8.440	78.34	±	13.21	60.96	±	5.084	56.46	±	3.221

793.6	PG_16:0/22:6	58.76	±	10.35	65.45	±	4.194	52.76	±	7.700	87.49	±	12.04	74.44	±	6.115	60.83	±	4.316
717.6	PG_16:1/16:1	209.8	±	25.49	415.9	±	67.20	306.4	±	32.82	229.6	±	27.96	460.4	±	11.51	383.1	±	12.58
747.6	PG_16:1/18:0	112.3	±	12.16	116.6	±	10.82	129.3	±	12.79	127.0	±	10.60	141.3	±	7.733	118.9	±	6.229
745.6	PG_16:1/18:1	1287	±	269.6	663.0	±	63.54	824.5	±	88.40	875.5	±	104.5	823.7	±	62.58	879.5	±	76.48
743.6	PG_16:1/18:2	433.2	±	135.7	334.3	±	53.68	339.6	±	92.89	525.3	±	106.9	462.7	±	68.25	532.1	±	79.37
741.6	PG_16:1/18:3	12.66	±	2.759	73.28	±	23.37	47.38	±	15.34	16.18	±	2.588	109.3	±	18.40	89.47	±	12.36
775.6	PG_16:1/20:0	29.38	±	5.311	115.5	±	21.83	84.36	±	11.27	32.48	±	7.343	148.7	±	10.99	114.0	±	6.931
773.6	PG_16:1/20:1	35.40	±	7.261	218.2	±	40.83	230.9	±	33.11	26.57	±	2.934	303.4	±	40.73	314.2	±	31.95
771.6	PG_16:1/20:2	25.02	±	4.176	108.9	±	18.27	109.3	±	30.03	23.54	±	2.316	177.1	±	41.94	205.6	±	37.12
769.6	PG_16:1/20:3	137.6	±	19.65	146.4	±	13.82	166.6	±	15.79	161.8	±	17.68	170.5	±	11.41	138.7	±	6.844
767.6	PG_16:1/20:4	84.29	±	22.41	348.2	±	42.92	213.2	±	52.00	110.1	±	20.19	357.7	±	35.36	325.0	±	12.02
765.6	PG_16:1/20:5	45.19	±	12.37	729.2	±	112.9	380.8	±	98.63	39.69	±	8.019	688.3	±	71.96	649.7	±	31.17
803.7	PG_16:1/22:0	57.24	±	10.31	74.34	±	15.34	64.16	±	11.78	64.63	±	9.434	111.4	±	11.64	95.27	±	3.996
801.7	PG_16:1/22:1	20.89	±	3.546	21.92	±	3.412	18.70	±	3.011	25.02	±	1.605	32.12	±	3.683	29.03	±	2.793
799.6	PG_16:1/22:2	15.82	±	2.284	15.51	±	2.173	17.92	±	1.32	16.72	±	2.324	23.87	±	2.137	19.72	±	1.562
797.6	PG_16:1/22:3	19.41	±	3.571	81.84	±	17.11	70.46	±	8.990	17.01	±	0.4986	116.7	±	4.859	74.62	±	5.072
795.6	PG_16:1/22:4	19.15	±	4.537	161.1	±	28.09	88.87	±	24.74	22.67	±	5.136	214.5	±	21.55	178.7	±	11.86
793.6	PG_16:1/22:5	47.25	±	10.80	324.6	±	59.42	186.0	±	52.79	63.66	±	16.09	438.8	±	45.28	413.2	±	17.82
791.6	PG_16:1/22:6	94.75	±	23.70	574.2	±	102.0	386.6	±	59.44	86.56	±	18.24	741.0	±	55.37	584.4	±	37.87
777.6	PG_18:0/18:0	22.22	±	3.823	21.28	±	2.160	20.62	±	1.669	28.37	±	2.676	45.25	±	4.439	32.00	±	1.853
775.6	PG_18:0/18:1	30.82	±	6.555	174.7	±	29.08	154.4	±	20.08	33.20	±	5.068	263.3	±	17.20	237.0	±	18.27
773.6	PG_18:0/18:2	12.22	±	1.582	18.13	±	1.821	16.11	±	2.369	18.62	±	3.459	28.78	±	2.796	25.58	±	3.131
771.6	PG_18:0/18:3	12.47	±	1.771	8.715	±	1.310	8.785	±	1.991	12.12	±	1.468	11.89	±	1.017	9.733	±	1.242
805.7	PG_18:0/20:0	36.54	±	7.801	16.91	±	1.480	19.68	±	2.133	43.30	±	4.654	22.90	±	1.256	18.80	±	0.5602
803.7	PG_18:0/20:1	13.66	±	2.515	11.72	±	1.028	10.80	±	1.436	20.05	±	2.717	12.26	±	1.461	13.39	±	0.5842
801.7	PG_18:0/20:2	8.060	±	1.435	4.580	±	1.009	4.367	±	1.595	10.58	±	1.379	6.560	±	1.564	4.941	±	1.847
799.6	PG_18:0/20:3	10.19	±	1.791	12.33	±	2.712	11.41	±	2.231	13.41	±	1.697	37.11	±	3.521	24.71	±	2.053
797.6	PG_18:0/20:4	9.540	±	0.9784	14.31	±	2.244	9.523	±	2.752	10.06	±	1.348	20.86	±	2.848	17.85	±	1.208
795.6	PG_18:0/20:5	19.42	±	2.353	43.52	±	4.327	28.17	±	6.031	23.25	±	2.967	49.52	±	5.877	44.94	±	2.460
833.7	PG_18:0/22:0	48.15	±	8.939	30.26	±	6.231	30.66	±	5.116	69.63	±	8.751	25.37	±	2.595	23.82	±	3.088
831.7	PG_18:0/22:1	6.360	±	1.557	4.930	±	1.202	4.668	±	0.6604	13.53	±	1.735	4.647	±	0.3621	1.887	±	1.194
829.7	PG_18:0/22:2	13.94	±	2.175	5.274	±	0.7269	8.106	±	1.178	16.08	±	1.717	4.543	±	1.090	5.115	±	0.4917
827.7	PG_18:0/22:3	7.890	±	2.290	3.662	±	0.7545	3.769	±	0.8276	10.84	±	1.314	6.287	±	0.4595	5.327	±	0.4625
825.7	PG_18:0/22:4	10.14	±	1.776	8.543	±	0.9725	7.810	±	1.887	14.64	±	2.225	12.92	±	1.444	11.90	±	1.174
823.7	PG_18:0/22:5	51.69	±	10.31	34.92	±	3.594	33.00	±	5.853	80.36	±	13.70	45.72	±	5.624	42.89	±	3.104
821.7	PG_18:0/22:6	81.37	±	13.24	56.64	±	4.537	51.33	±	8.128	103.7	±	14.36	53.86	±	5.467	50.90	±	2.683

773.6	PG_18:1/18:1	65.05	±	15.62	595.4	±	118.2	613.9	±	82.90	34.79	±	3.025	834.3	±	126.8	888.6	±	105.5
771.6	PG_18:1/18:2	32.67	±	8.350	358.0	±	81.59	329.7	±	104.1	23.96	±	3.681	643.6	±	123.3	690.1	±	119.5
769.6	PG_18:1/18:3	5.610	±	1.892	51.11	±	14.75	39.33	±	13.17	9.324	±	1.875	104.0	±	18.37	85.78	±	11.53
803.7	PG_18:1/20:0	19.66	±	3.662	86.55	±	20.70	77.16	±	13.69	23.31	±	3.975	169.3	±	15.03	129.8	±	8.844
801.7	PG_18:1/20:1	13.97	±	2.563	130.7	±	23.86	143.2	±	23.02	14.59	±	1.634	246.8	±	28.22	264.8	±	33.47
799.6	PG_18:1/20:2	9.353	±	2.261	86.90	±	16.48	86.81	±	21.00	9.544	±	1.805	174.9	±	28.44	190.1	±	33.19
797.6	PG_18:1/20:3	16.75	±	4.240	196.4	±	42.97	154.7	±	20.04	12.29	±	2.199	293.1	±	19.02	177.8	±	11.82
795.6	PG_18:1/20:4	15.12	±	5.445	292.8	±	58.52	175.0	±	48.71	15.27	±	3.434	456.9	±	47.90	370.5	±	23.78
793.6	PG_18:1/20:5	24.40	±	6.099	531.7	±	102.8	303.9	±	93.61	31.95	±	7.033	758.3	±	84.66	719.7	±	32.50
831.7	PG_18:1/22:0	25.33	±	6.058	46.84	±	8.156	41.43	±	9.400	27.08	±	3.098	92.98	±	7.223	87.91	±	6.408
829.7	PG_18:1/22:1	7.281	±	1.930	23.94	±	3.713	19.18	±	3.463	9.289	±	1.174	42.71	±	3.631	30.98	±	2.988
827.7	PG_18:1/22:2	5.628	±	1.894	22.45	±	4.618	17.63	±	2.059	9.609	±	2.961	34.33	±	3.463	28.63	±	1.571
825.7	PG_18:1/22:3	6.900	±	1.736	59.51	±	13.80	46.57	±	6.419	6.210	±	1.441	108.4	±	4.163	74.88	±	3.787
823.7	PG_18:1/22:4	7.689	±	2.603	105.7	±	24.68	64.02	±	18.44	11.36	±	1.742	199.1	±	20.48	168.5	±	8.835
821.7	PG_18:1/22:5	21.33	±	3.673	183.4	±	41.49	117.4	±	34.82	37.65	±	7.577	354.7	±	40.51	330.5	±	16.93
679.5	PG_a16:0/14:0	39.01	±	5.234	36.68	±	3.215	40.84	±	2.228	50.04	±	7.059	48.04	±	4.440	38.92	±	2.168
707.6	PG_a16:0/16:0	14.82	±	2.802	10.44	±	0.8365	14.95	±	5.790	22.20	±	3.112	9.519	±	0.8512	9.759	±	1.301
735.6	PG_a16:0/18:0	15.35	±	2.612	6.508	±	0.6168	12.81	±	5.783	26.68	±	3.371	6.574	±	0.4691	8.789	±	0.9315
735.6	PG_a18:0/16:0	12.12	±	2.120	12.29	±	0.9862	11.91	±	5.073	19.04	±	2.988	12.74	±	1.489	10.47	±	1.597
763.6	PG_a18:0/18:0	10.73	±	1.998	7.513	±	0.9593	8.461	±	1.976	16.78	±	2.166	7.600	±	0.5586	7.517	±	1.037
735.6	PG_a20:0/14:0	75.24	±	15.90	76.15	±	12.80	73.83	±	21.06	116.5	±	26.37	76.02	±	13.14	61.74	±	7.627
763.6	PG_a20:0/16:0	12.46	±	2.151	20.45	±	1.782	17.57	±	3.400	18.38	±	2.527	42.93	±	3.877	34.95	±	3.749
791.6	PG_a20:0/18:0	11.69	±	2.017	11.06	±	0.7681	8.377	±	1.066	17.33	±	2.370	11.37	±	1.422	10.28	±	0.736
677.5	PG_p16:0/14:0	14.04	±	4.350	18.61	±	1.475	17.79	±	5.208	31.32	±	6.102	31.07	±	6.028	31.19	±	4.682
705.6	PG_p16:0/16:0	20.43	±	3.516	15.70	±	2.234	14.96	±	2.909	31.51	±	4.946	10.66	±	1.808	9.896	±	0.8863
703.6	PG_p16:0/16:1	24.99	±	3.947	147.8	±	21.21	33.35	±	5.763	32.02	±	4.708	42.68	±	4.215	27.65	±	1.431
733.6	PG_p16:0/18:0	22.02	±	3.531	14.52	±	1.554	16.68	±	3.726	35.65	±	5.174	9.623	±	1.134	10.29	±	0.7895
733.6	PG_p18:0/16:0	17.93	±	3.307	19.79	±	0.9314	16.51	±	3.188	30.29	±	4.138	17.43	±	1.755	17.32	±	1.242
731.6	PG_p18:0/16:1	25.02	±	6.297	75.67	±	12.76	36.99	±	9.248	42.52	±	10.86	54.11	±	4.637	48.34	±	2.379
761.6	PG_p18:0/18:0	18.62	±	2.237	12.56	±	1.308	11.89	±	2.014	29.03	±	5.360	8.876	±	0.9712	9.371	±	0.6619
759.6	PG_p18:0/18:1	9.748	±	2.562	110.6	±	26.22	41.82	±	11.40	15.29	±	3.338	80.64	±	6.070	64.27	±	1.895
733.6	PG_p20:0/14:0	10.17	±	2.218	8.447	±	1.125	7.977	±	2.878	18.18	±	2.826	13.16	±	1.994	12.65	±	1.846
761.6	PG_p20:0/16:0	14.60	±	1.808	18.78	±	2.023	14.47	±	2.695	19.42	±	3.036	25.30	±	3.394	20.81	±	2.647
759.6	PG_p20:0/16:1	24.68	±	2.824	52.99	±	7.280	50.30	±	7.689	20.51	±	2.528	62.44	±	4.261	62.37	±	4.667
789.6	PG_p20:0/18:0	10.64	±	1.571	9.826	±	0.9011	6.344	±	1.453	17.60	±	2.025	13.15	±	0.8710	11.96	±	0.954
787.6	PG_p20:0/18:1	11.70	±	2.828	52.00	±	9.677	47.62	±	8.598	10.97	±	1.682	77.81	±	5.232	81.84	±	10.90

543.4	LPI_14:0_	6.944	±	1.782	5.571	±	1.299	5.214	±	1.281	11.46	±	1.493	3.480	±	0.7041	2.161	±	0.9698
569.4	LPI_16:1_	61.73	±	13.02	43.81	±	8.204	51.82	±	8.581	88.07	±	17.12	64.95	±	4.718	61.40	±	1.542
571.4	LPI_16:0_	91.87	±	16.72	55.19	±	4.886	58.53	±	8.703	127.3	±	16.00	42.62	±	1.887	42.35	±	2.358
597.4	LPI_18:1_	36.80	±	7.058	30.73	±	4.517	36.34	±	7.759	66.66	±	12.21	54.65	±	4.466	59.89	±	1.846
599.4	LPI_18:0_	95.51	±	14.73	51.23	±	6.379	55.30	±	7.375	134.4	±	17.98	33.57	±	1.368	36.84	±	2.037
753.6	PI_14:0/14:0	10.05	±	1.133	7.214	±	0.8114	3.987	±	1.428	10.48	±	1.766	5.775	±	1.904	5.718	±	1.521
779.6	PI_14:0/16:1	16.69	±	2.444	135.9	±	30.16	75.62	±	16.54	25.92	±	5.227	163.1	±	19.37	129.6	±	9.399
781.6	PI_14:0/16:0	14.85	±	1.496	25.66	±	3.121	18.01	±	2.786	22.18	±	3.246	27.65	±	2.896	22.37	±	1.604
805.7	PI_16:1/16:1	116.1	±	17.11	534.1	±	106.0	290.8	±	31.06	113.3	±	19.22	476.8	±	34.22	312.0	±	7.632
807.7	PI_14:0/18:1	16.28	±	2.566	156.6	±	36.19	84.93	±	12.67	17.94	±	3.702	201.9	±	18.29	154.7	±	6.340
807.7	PI_16:0/16:1	121.0	±	20.84	617.0	±	111.5	387.9	±	52.50	110.6	±	16.06	556.5	±	36.83	415.9	±	22.51
809.7	PI_14:0/18:0	20.98	±	3.158	16.29	±	1.328	15.22	±	2.710	24.54	±	2.946	16.45	±	1.449	13.13	±	0.926
809.7	PI_16:0/16:0	44.42	±	5.764	76.01	±	8.150	56.75	±	3.041	67.55	±	10.29	67.77	±	3.837	52.52	±	3.753
831.7	PI_16:0/18:3	10.75	±	3.207	10.40	±	0.8318	5.714	±	0.3687	9.574	±	1.402	54.03	±	9.580	7.601	±	0.9943
831.7	PI_16:1/18:2	24.78	±	6.285	34.26	±	2.500	20.98	±	3.672	20.86	±	3.173	44.27	±	2.265	39.11	±	2.814
833.7	PI_16:1/18:1	75.96	±	14.32	601.4	±	142.6	315.8	±	19.67	108.2	±	23.09	673.7	±	31.21	464.7	±	12.63
833.7	PI_16:0/18:2	22.26	±	3.643	37.33	±	4.377	16.35	±	0.4575	26.88	±	4.928	24.37	±	1.005	16.62	±	2.033
835.7	PI_16:1/18:0	100.8	±	16.69	259.8	±	49.09	170.4	±	6.740	142.1	±	22.19	271.7	±	6.788	210.6	±	6.478
835.7	PI_16:0/18:1	131.0	±	40.41	1493	±	379.6	828.0	±	80.28	94.34	±	20.47	1701	±	98.23	1166	±	53.02
837.7	PI_14:0/20:0	13.30	±	2.848	7.260	±	0.7578	8.159	±	2.084	17.65	±	1.736	4.789	±	1.101	6.704	±	1.650
837.7	PI_16:0/18:0	52.37	±	6.019	95.28	±	15.01	66.04	±	2.318	74.07	±	11.46	101.2	±	4.853	79.51	±	5.201
849.7	PI_p18:0/18:0	18.01	±	3.987	123.6	±	21.68	73.76	±	11.60	24.98	±	4.519	158.7	±	15.08	111.2	±	8.126
849.7	PI_p20:0/16:0	38.25	±	7.363	312.1	±	67.40	194.9	±	39.24	51.50	±	7.462	534.2	±	47.83	386.4	±	37.80
857.7	PI_16:1/20:3	14.72	±	2.985	15.27	±	2.777	9.862	±	1.320	15.22	±	2.104	15.34	±	1.292	16.06	±	1.946
857.7	PI_16:0/20:4	11.63	±	1.797	9.535	±	1.133	9.728	±	0.5850	14.08	±	2.314	10.64	±	1.972	8.160	±	0.7783
857.7	PI_18:1/18:3	3.732	±	1.256	18.50	±	3.564	16.09	±	2.556	4.610	±	0.9968	74.58	±	10.46	21.17	±	2.086
859.7	PI_16:1/20:2	16.89	±	3.679	20.00	±	3.281	14.70	±	2.786	19.78	±	1.896	24.56	±	2.188	21.81	±	1.160
859.7	PI_16:0/20:3	6.961	±	1.075	3.643	±	1.245	5.228	±	0.6344	11.44	±	2.198	4.710	±	0.9817	3.289	±	1.098
859.7	PI_18:0/18:3	5.819	±	1.332	6.833	±	0.5662	3.900	±	0.8732	9.815	±	2.040	44.39	±	10.15	2.598	±	0.8331
859.7	PI_18:1/18:2	5.828	±	1.463	24.48	±	2.283	16.15	±	3.420	8.017	±	2.121	38.21	±	1.857	28.86	±	2.540
861.7	PI_16:1/20:1	11.87	±	2.29	32.01	±	6.180	20.75	±	5.635	19.31	±	3.970	44.50	±	5.073	33.19	±	4.608
861.7	PI_16:0/20:2	7.445	±	1.115	5.848	±	1.254	5.647	±	0.7554	12.57	±	1.984	7.564	±	0.7399	7.259	±	0.5544
861.7	PI_18:0/18:2	13.10	±	2.764	12.12	±	1.335	10.09	±	1.221	18.37	±	2.134	14.70	±	1.296	8.487	±	0.9896
861.7	PI_18:1/18:1	12.12	±	2.739	145.6	±	38.64	77.40	±	12.03	14.38	±	3.742	227.0	±	8.676	167.0	±	6.518
863.7	PI_14:0/22:1	19.68	±	4.252	10.05	±	1.067	13.69	±	1.238	32.42	±	5.564	10.85	±	1.334	11.04	±	1.207
863.7	PI_16:1/20:0	116.3	±	26.62	72.86	±	6.976	81.60	±	18.52	151.1	±	30.22	119.0	±	8.510	100.9	±	4.789

863.7	PI_16:0/20:1	13.70	±	2.141	14.75	±	0.6582	12.63	±	1.436	18.86	±	2.386	14.33	±	1.804	14.17	±	2.284
863.7	PI_18:0/18:1	59.23	±	11.36	471.4	±	140.4	257.9	±	17.14	61.07	±	10.47	664.0	±	21.76	467.0	±	11.75
865.7	PI_16:0/20:0	147.1	±	23.97	71.09	±	8.850	83.77	±	13.11	211.1	±	27.29	54.58	±	6.651	61.96	±	3.809
865.7	PI_18:0/18:0	56.49	±	7.249	56.76	±	8.011	48.81	±	2.510	84.45	±	12.24	76.73	±	5.432	58.95	±	5.287
883.7	PI_16:1/22:4	10.35	±	2.375	14.41	±	1.391	14.41	±	4.219	16.01	±	3.736	16.81	±	1.969	19.96	±	1.212
883.7	PI_16:0/22:5	8.128	±	1.657	6.754	±	0.6123	6.156	±	0.9127	10.82	±	1.296	6.660	±	0.5817	6.793	±	0.5478
883.7	PI_18:1/20:4	3.244	±	1.530	13.16	±	2.278	11.98	±	1.831	8.225	±	2.119	23.21	±	2.351	20.00	±	2.136
883.7	PI_18:0/20:5	19.05	±	1.945	11.55	±	1.389	12.34	±	1.276	27.40	±	4.793	9.352	±	1.004	10.81	±	1.259
885.7	PI_16:1/22:3	10.79	±	1.926	15.20	±	1.681	12.36	±	3.376	14.97	±	3.369	13.02	±	2.291	16.22	±	1.267
885.7	PI_18:1/20:3	3.144	±	1.064	9.086	±	2.516	9.124	±	1.493	3.695	±	1.209	15.39	±	1.685	12.73	±	0.4656
885.7	PI_18:0/20:4	11.64	±	0.9665	7.422	±	1.029	7.463	±	1.559	16.17	±	3.132	3.143	±	1.419	5.279	±	1.261
887.7	PI_16:1/22:2	6.948	±	1.884	12.06	±	1.778	10.11	±	1.487	7.331	±	0.9685	11.97	±	1.505	13.14	±	1.111
887.7	PI_18:1/20:2	2.557	±	1.195	12.79	±	2.502	11.10	±	2.955	5.006	±	1.762	21.49	±	1.716	16.99	±	1.447
887.7	PI_18:0/20:3	6.862	±	0.7873	1.612	±	1.050	5.301	±	0.5300	10.61	±	1.742	1.982	±	0.8916	1.902	±	1.241
889.7	PI_16:1/22:1	178.4	±	42.29	83.11	±	16.41	104.2	±	17.07	240.1	±	54.31	177.1	±	19.41	154.8	±	8.307
889.7	PI_16:0/22:2	6.773	±	1.202	3.993	±	0.8755	5.975	±	1.464	8.804	±	1.689	3.595	±	1.176	4.881	±	1.017
889.7	PI_18:1/20:1	2.767	±	1.251	18.42	±	3.537	16.34	±	4.376	6.196	±	1.750	33.65	±	4.319	23.26	±	1.447
889.7	PI_18:0/20:2	7.020	±	0.6879	3.305	±	1.069	0.748	±	0.748	10.66	±	2.026	4.135	±	0.8724	4.182	±	0.9236
891.7	PI_16:1/22:0	33.17	±	5.327	23.83	±	4.015	23.03	±	4.384	41.16	±	6.380	39.14	±	4.448	34.72	±	2.742
891.7	PI_16:0/22:1	280.8	±	52.35	110.5	±	9.270	159.1	±	22.46	398.9	±	46.86	122.8	±	12.18	119.8	±	4.829
891.7	PI_18:1/20:0	67.56	±	11.05	51.86	±	5.253	60.88	±	13.94	104.3	±	16.64	95.06	±	7.534	100.6	±	4.552
891.7	PI_18:0/20:1	12.65	±	1.949	7.416	±	1.012	6.198	±	0.4065	18.87	±	2.273	7.926	±	0.6943	7.232	±	0.7897
893.7	PI_16:0/22:0	31.16	±	5.835	21.43	±	1.577	23.96	±	3.218	43.16	±	5.583	23.89	±	2.897	23.11	±	2.146
893.7	PI_18:0/20:0	164.5	±	23.60	56.48	±	9.561	74.33	±	9.662	209.1	±	29.17	41.14	±	3.758	47.81	±	4.343
911.8	PI_18:1/22:4	1.677	±	1.066	18.37	±	2.943	10.15	±	2.529	3.856	±	1.224	22.83	±	3.833	23.34	±	1.916
911.8	PI_18:0/22:5	6.501	±	0.7114	3.832	±	1.296	4.084	±	1.470	11.15	±	1.915	3.560	±	0.7428	3.399	±	1.117
913.8	PI_18:1/22:3	B.Q.L.	±	B.Q.L.	14.56	±	2.520	5.865	±	2.317	3.014	±	1.583	18.94	±	2.625	16.41	±	1.464
915.8	PI_18:1/22:2	2.389	±	1.069	9.642	±	2.476	7.244	±	0.5565	1.986	±	1.293	14.43	±	1.139	11.27	±	1.207
917.8	PI_18:1/22:1	99.96	±	18.52	54.07	±	8.563	82.60	±	12.39	177.9	±	28.84	141.0	±	18.46	135.2	±	5.260
412.3	LPS_10:0	19.95	±	13.62	17.99	±	5.576	18.47	±	5.294	20.37	±	5.889	10.35	±	3.742	13.65	±	4.569
440.3	LPS_12:0	75.77	±	8.982	38.82	±	9.719	59.66	±	8.376	82.70	±	18.67	28.77	±	6.760	32.61	±	11.40
468.3	LPS_14:0	201.3	±	36.31	209.4	±	35.29	192.9	±	59.66	259.2	±	22.27	146.6	±	25.93	199.5	±	20.12
494.3	LPS_16:1	1708	±	347.2	1124	±	206.3	1572	±	367.2	1946	±	265.5	1820	±	191.1	2528	±	152.8
496.3	LPS_16:0	2152	±	235.0	1452	±	285.8	1476	±	179.4	2577	±	109.5	844.3	±	65.68	1204	±	89.91
520.4	LPS_18:2	42.07	±	6.694	47.77	±	11.91	27.23	±	5.074	40.46	±	7.348	26.96	±	6.040	20.42	±	8.751
522.4	LPS_18:1	1184	±	167.6	1049	±	159.1	1371	±	375.4	1700	±	173.2	1977	±	261.1	2446	±	201.8

524.4	LPS_18:0	2445	±	204.3	1569	±	333.4	1671	±	182.5	3085	±	199.5	918.7	±	79.18	1196	±	114.6
552.4	LPS_20:0	41.19	±	14.03	25.7	±	10.70	35.08	±	6.418	65.87	±	10.31	27.87	±	4.544	17.12	±	4.517
650.5	PS_12:0/14:0	358.3	±	42.17	294.8	±	47.09	164.2	±	21.44	326.6	±	44.00	155.4	±	22.58	151.0	±	19.75
730.6	PS_14:0/18:2	124.7	±	20.94	186.3	±	23.39	143.1	±	12.67	118.8	±	23.09	156.4	±	28.32	143.9	±	27.11
730.6	PS_16:1/16:1	2622	±	415.5	8531	±	1112	4258	±	1267	4412	±	942.6	5694	±	834.8	5948	±	569.1
732.6	PS_14:0/18:1	321.1	±	30.82	2202	±	405.4	614.4	±	106.7	307.5	±	22.36	853.8	±	149.9	633.8	±	68.57
732.6	PS_16:0/16:1	1018	±	72.37	1949	±	239.5	1192	±	170.6	1202	±	172.2	1234	±	155.8	1222	±	94.71
734.6	PS_16:0/16:0	418.7	±	27.64	385.5	±	43.63	350.5	±	59.22	615.8	±	55.86	365.2	±	48.53	427.3	±	42.92
758.6	PS_14:0/20:2	431.9	±	64.70	122.9	±	26.82	195.7	±	24.40	291.4	±	34.14	79.9	±	12.17	107.1	±	19.84
758.6	PS_16:1/18:1	2074	±	215.0	8222	±	1069	5822	±	1082	1561	±	199.5	7462	±	741.1	8276	±	305.7
758.6	PS_16:0/18:2	390.7	±	29.25	653.0	±	60.16	491.4	±	78.03	421.3	±	59.57	505.0	±	59.41	506.2	±	41.55
760.6	PS_14:0/20:1	324.4	±	100.8	166.4	±	28.99	154.7	±	25.53	278.0	±	58.74	226.0	±	42.51	230.7	±	22.90
760.6	PS_16:0/18:1	732.6	±	40.22	1762	±	204.2	1275	±	183.2	667.7	±	52.14	1519	±	145.1	1558	±	37.79
762.6	PS_16:0/18:0	463.2	±	19.38	1096	±	164.2	734.9	±	97.15	518.1	±	32.45	2185	±	190.3	2119	±	173.7
776.6	PS_a18:0/18:0	317.8	±	42.65	589.7	±	115.7	756.6	±	59.45	278.8	±	30.68	1421	±	133.5	1225	±	70.74
782.6	PS_14:0/22:4	78.22	±	14.32	78.18	±	5.725	63.51	±	13.61	67.18	±	11.75	59.35	±	6.688	35.80	±	9.702
782.6	PS_16:1/20:3	361.9	±	33.80	1469	±	298.3	875.6	±	113.6	356.9	±	39.12	1347	±	189.2	1269	±	86.89
782.6	PS_16:0/20:4	249.3	±	30.00	2041	±	344.6	723.9	±	255.6	229.1	±	37.11	1542	±	243.8	1434	±	109.1
782.6	PS_18:1/18:3	271.7	±	63.81	7092	±	1446	3091	±	976.5	407.2	±	60.93	6407	±	980.1	5806	±	374.0
784.6	PS_14:0/22:3	112.3	±	25.13	54.06	±	9.570	64.31	±	14.68	103.6	±	14.10	40.11	±	5.923	35.69	±	4.813
784.6	PS_16:1/20:2	652.8	±	137.5	583.2	±	65.07	499.5	±	37.52	437.2	±	90.97	480.6	±	46.67	458.1	±	37.57
784.6	PS_16:0/20:3	124.2	±	14.10	297.5	±	71.40	148.1	±	19.39	148.4	±	16.84	245.5	±	34.61	243.2	±	19.61
784.6	PS_18:1/18:2	302.8	±	42.21	2273	±	400.3	1484	±	358.1	298.5	±	48.33	2644	±	318.8	2618	±	102.1
786.6	PS_16:1/20:1	862.2	±	58.62	1952	±	186.7	1971	±	269.8	893.2	±	121.9	1830	±	214.7	2575	±	224.1
786.6	PS_16:0/20:2	131.6	±	13.12	203.4	±	27.44	147.2	±	15.96	155.3	±	28.59	490.7	±	88.13	539.8	±	39.40
786.6	PS_18:0/18:2	363.7	±	48.59	292.3	±	32.77	209.2	±	23.42	370.7	±	25.01	349.7	±	59.97	346.9	±	43.01
786.6	PS_18:1/18:1	792.5	±	170.1	3571	±	613.0	4146	±	579.0	354.0	±	40.59	4274	±	528.0	5682	±	530.6
788.6	PS_16:0/20:1	273.8	±	58.99	249.5	±	15.76	268.5	±	30.1	292.9	±	35.08	223.9	±	20.34	250.4	±	10.53
788.6	PS_18:0/18:1	683.6	±	61.76	2190	±	320.4	1669	±	126.4	614.4	±	72.43	2458	±	183.5	2324	±	147.2
790.6	PS_18:0/18:0	501.9	±	49.13	680.7	±	57.48	430.7	±	45.98	493.8	±	76.16	854.7	±	115.6	838.8	±	65.47
810.7	PS_18:0/20:4	194.9	±	22.97	246.1	±	33.00	148.7	±	38.21	226.2	±	14.37	278.8	±	43.03	234.8	±	27.12
812.7	PS_16:0/22:3	173.8	±	31.85	222.3	±	21.55	174.3	±	40.21	280.8	±	41.63	162.0	±	10.28	150.3	±	15.25
812.7	PS_18:1/20:2	149.6	±	36.15	634.9	±	70.66	425.2	±	15.72	138.7	±	30.98	654.8	±	32.62	626.0	±	53.21
814.7	PS_18:1/20:1	256.5	±	47.52	1563	±	206.0	1423	±	253.7	180.3	±	33.17	1901	±	142.5	2335	±	185.6
814.7	PS_18:2/20:0	109.8	±	31.35	1452	±	93.45	874.1	±	83.03	84.27	±	6.545	1149	±	128.6	836.3	±	32.97
816.7	PS_18:0/20:1	229.6	±	44.65	144.9	±	15.21	153.3	±	26.17	264.7	±	36.97	154.4	±	19.84	192.9	±	28.49

838.7	PS_18:1/22:3	202.2	±	25.96	1984	±	230.6	1263	±	331.4	260.5	±	48.91	1807	±	117.9	2282	±	114.7
838.7	PS_18:0/22:4	225.3	±	46.29	188.1	±	12.93	150.7	±	30.14	246.0	±	25.74	211.5	±	52.64	163.4	±	6.755
840.7	PS_18:0/22:3	143.9	±	27.60	110.5	±	19.55	112.3	±	13.79	197.3	±	25.61	75.48	±	10.52	109.2	±	9.780

Supplementary Table 4: Concentrations (in pmol/mg protein) of cardiolipins (CL), and monolysocardiolipins (MLCL) in Aac2 and ArANT expressing yeast cells, Percoll-purified mitochondria and mitoplasts. N.D.: not determined. B.Q.L.: below quantitative limit. For CL determination, all the fragments that would comprise the acyl chains were summed up and isomeric composition for the molecular species as the brutto nomenclature (carbon:double bond) is given. Results shown are Mean +/- S.E.M.

		**Aac2**			**Artemia-ANT**		
**[M-2H]$^{2-}$**	**Species**	**Yeast Cell**	**Mitochondria**	**Mitoplasts**	**Yeast Cell**	**Mitochondria**	**Mitoplasts**
658.6	CL_62:3	67.27 ± 12.96	932.0 ± 152.8	527.4 ± 55.40	59.28 ± 14.16	970.0 ± 77.64	708.1 ± 40.22
659.6	CL_62:2	60.34 ± 17.26	961.9 ± 189.8	503.9 ± 52.99	51.84 ± 14.74	869.3 ± 69.11	659.2 ± 41.42
670.6	CL_64:5	29.53 ± 7.773	59.29 ± 8.837	52.8 ± 24.87	30.92 ± 5.869	37.89 ± 7.084	32.91 ± 5.487
671.6	CL_64:4	47.70 ± 9.157	796.6 ± 98.81	450.0 ± 11.98	44.79 ± 11.26	746.4 ± 54.25	521.7 ± 37.24
672.6	CL_64:3	97.46 ± 15.36	1164 ± 209.4	640.3 ± 45.73	101.8 ± 29.00	1151 ± 79.14	890.1 ± 73.02
673.6	CL_64:2	53.41 ± 7.607	674.6 ± 99.58	358.4 ± 31.85	47.95 ± 9.275	657.2 ± 54.07	493.5 ± 45.01
674.6	CL_64:1	33.58 ± 5.852	178.3 ± 33.73	104.2 ± 11.94	46.49 ± 7.820	130.7 ± 10.76	101.5 ± 7.496
683.6	CL_66:6	10.59 ± 3.024	52.20 ± 6.418	38.20 ± 6.699	20.01 ± 5.397	44.95 ± 3.705	25.50 ± 3.627
684.6	CL_66:5	62.84 ± 9.208	153.1 ± 17.26	148.4 ± 24.79	48.15 ± 18.51	107.9 ± 15.13	88.06 ± 13.54
685.6	CL_66:4	224.8 ± 36.47	3747 ± 643.3	1837 ± 152.2	172.0 ± 45.56	4193 ± 324.8	2834 ± 216.6
686.6	CL_66:3	235.8 ± 42.47	6074 ± 2717	1373 ± 94.30	183.8 ± 46.55	2528 ± 192.8	5110 ± 3220
687.6	CL_66:2	97.02 ± 22.44	1035 ± 169.5	548.9 ± 43.93	98.90 ± 27.15	935.6 ± 88.34	716.8 ± 69.26
688.6	CL_66:1	50.61 ± 5.433	168.2 ± 19.43	130.8 ± 11.93	55.35 ± 10.92	162.6 ± 22.36	138.7 ± 11.70
695.6	CL_68:8	14.33 ± 2.418	19.33 ± 4.957	25.59 ± 6.068	23.89 ± 6.669	26.96 ± 5.383	23.60 ± 5.045
696.6	CL_68:7	33.12 ± 5.003	66.45 ± 6.172	61.54 ± 10.52	47.83 ± 9.368	44.37 ± 5.345	35.66 ± 7.317
697.6	CL_68:6	23.23 ± 4.696	197.1 ± 14.08	113.9 ± 10.07	23.43 ± 3.038	92.01 ± 19.75	102.6 ± 13.05
698.6	CL_68:5	55.24 ± 6.787	522.8 ± 50.14	304.7 ± 22.58	63.82 ± 13.66	374.5 ± 26.22	335.2 ± 22.17
699.6	CL_68:4	533.6 ± 87.31	7669 ± 1591	3849 ± 450.2	430.9 ± 101.5	10370 ± 903.8	7227 ± 661.7
700.7	CL_68:3	463.4 ± 61.28	5662 ± 1072	2767 ± 317.9	415.0 ± 99.62	6309 ± 681.0	4469 ± 367.7
701.7	CL_68:2	195.8 ± 30.32	1746 ± 349.8	1002 ± 135.5	160.5 ± 33.40	1915 ± 229.8	1406 ± 126.6
702.7	CL_68:1	145.4 ± 26.19	280.8 ± 44.39	202.8 ± 26.18	119.3 ± 33.23	276.4 ± 22.50	258.1 ± 21.69
703.7	CL_68:0	33.74 ± 3.350	37.88 ± 7.097	44.95 ± 11.88	32.51 ± 7.440	43.06 ± 9.197	34.18 ± 6.146
704.7	CL_70:13	26.72 ± 5.105	26.58 ± 5.865	37.96 ± 12.87	39.16 ± 16.07	20.72 ± 5.884	16.50 ± 4.300
706.7	CL_70:11	27.15 ± 2.925	45.71 ± 7.852	48.71 ± 10.54	40.85 ± 7.676	37.11 ± 4.851	29.01 ± 5.771
707.7	CL_70:10	11.56 ± 2.195	32.64 ± 8.732	21.85 ± 3.155	20.92 ± 6.116	27.48 ± 8.159	31.03 ± 4.947
708.7	CL_70:9	39.59 ± 9.493	37.37 ± 6.948	45.90 ± 12.36	42.15 ± 9.854	29.00 ± 2.785	32.07 ± 7.412
709.7	CL_70:8	17.27 ± 2.808	63.64 ± 5.444	38.16 ± 4.375	16.73 ± 4.097	40.48 ± 6.942	31.84 ± 3.814
710.7	CL_70:7	31.91 ± 5.564	93.66 ± 14.76	48.39 ± 9.195	46.40 ± 9.255	37.08 ± 8.187	39.72 ± 5.760
711.7	CL_70:6	27.82 ± 3.778	248.3 ± 27.42	134.7 ± 11.60	37.24 ± 7.753	136.4 ± 26.85	97.02 ± 11.08
712.7	CL_70:5	76.76 ± 11.03	567.7 ± 82.67	308.1 ± 14.67	72.79 ± 15.42	512.9 ± 48.89	373.2 ± 43.61

713.7	CL_70:4	473.9	±	82.03	5447	±	1146	2844	±	453.3	432.2	±	90.16	8606	±	851.5	5973	±	619.4
714.7	CL_70:3	335.7	±	48.64	4700	±	2634	1515	±	248.0	328.2	±	55.55	3043	±	311.9	6627	±	4152
715.7	CL_70:2	129.6	±	11.77	683.4	±	116.4	414.5	±	64.90	117.1	±	20.12	927.3	±	113.4	697.4	±	61.38
716.7	CL_70:1	92.37	±	9.467	154.6	±	20.39	188.7	±	32.74	106.4	±	16.79	223.8	±	20.46	184.7	±	17.55
717.7	CL_70:0	48.67	±	4.499	75.24	±	8.738	82.30	±	16.03	47.85	±	6.607	88.02	±	9.156	75.39	±	13.00
719.7	CL_72:12	13.20	±	2.621	40.49	±	4.233	26.95	±	4.885	29.66	±	7.608	28.24	±	6.455	32.41	±	8.167
720.7	CL_72:11	34.72	±	5.639	50.56	±	8.711	45.08	±	10.07	43.45	±	10.40	37.98	±	4.005	36.22	±	6.545
721.7	CL_72:10	15.61	±	2.757	27.29	±	4.046	34.29	±	13.81	23.88	±	6.187	36.96	±	6.432	23.64	±	4.264
722.7	CL_72:9	29.10	±	5.001	53.81	±	5.419	67.16	±	17.92	46.92	±	9.005	43.84	±	11.30	37.33	±	4.578
723.7	CL_72:8	18.38	±	1.676	81.29	±	10.03	51.3	±	7.194	22.78	±	3.144	55.29	±	7.150	34.98	±	5.615
724.7	CL_72:7	30.25	±	4.377	111.3	±	12.40	85.63	±	8.863	45.50	±	8.307	74.71	±	7.869	62.50	±	10.44
725.7	CL_72:6	23.38	±	3.565	146.3	±	22.51	92.64	±	7.860	27.04	±	9.526	140.7	±	13.61	118.4	±	15.26
726.7	CL_72:5	52.70	±	5.822	222.8	±	23.79	123.2	±	13.09	54.01	±	12.23	102.3	±	15.66	103.4	±	8.182
727.7	CL_72:4	146.1	±	22.53	1344	±	289.9	733.5	±	144.1	153.6	±	31.02	1628	±	180.2	1243	±	114.7
728.7	CL_72:3	88.33	±	15.24	541.0	±	105.1	318.1	±	60.57	109.5	±	18.05	870.5	±	102.0	590.8	±	53.08
729.7	CL_72:2	24.02	±	3.221	116.1	±	18.02	84.63	±	17.70	36.54	±	9.047	154.6	±	10.59	130.9	±	14.49
730.7	CL_72:1	39.84	±	7.170	59.74	±	8.283	60.44	±	10.55	60.73	±	12.14	51.18	±	8.939	40.78	±	4.265
731.7	CL_72:0	17.70	±	1.932	18.32	±	3.165	21.57	±	5.773	21.04	±	4.434	22.19	±	6.333	23.72	±	5.243
732.7	CL_74:13	24.50	±	4.775	25.11	±	2.251	27.85	±	7.232	35.89	±	8.835	24.43	±	4.153	22.32	±	4.032
734.7	CL_74:11	35.35	±	11.92	27.86	±	5.514	30.30	±	7.381	50.43	±	13.15	19.55	±	4.914	22.19	±	4.156
735.7	CL_74:10	15.52	±	2.295	19.42	±	3.111	24.11	±	6.646	18.86	±	3.454	22.75	±	6.698	17.87	±	3.449
736.7	CL_74:9	38.32	±	7.312	32.02	±	5.353	40.98	±	12.06	51.46	±	14.01	32.31	±	3.707	32.88	±	4.391
737.7	CL_74:8	19.56	±	6.336	36.08	±	9.203	28.26	±	6.922	30.69	±	4.979	34.71	±	6.104	30.70	±	5.039
738.7	CL_74:7	28.02	±	3.141	44.57	±	6.337	48.13	±	9.480	51.38	±	10.03	28.35	±	9.791	25.91	±	3.136
739.7	CL_74:6	16.58	±	1.866	23.91	±	9.598	26.85	±	6.334	23.57	±	5.183	17.91	±	5.240	23.75	±	3.546
740.7	CL_74:5	22.89	±	4.052	29.48	±	9.847	36.50	±	10.89	44.48	±	7.479	26.39	±	2.836	25.58	±	6.905
741.7	CL_74:4	16.87	±	4.239	22.70	±	5.579	21.93	±	7.072	24.72	±	4.794	31.15	±	6.888	21.84	±	3.465
742.7	CL_74:3	36.29	±	3.654	39.97	±	6.649	59.59	±	17.02	49.23	±	9.948	38.03	±	6.926	32.90	±	6.793
743.7	CL_74:2	14.70	±	2.609	27.70	±	3.074	23.59	±	8.587	24.64	±	4.215	33.91	±	5.156	25.10	±	3.416
743.7	CL_74:1	18.90	±	1.963	27.27	±	1.848	31.34	±	10.72	23.12	±	4.613	31.82	±	7.352	31.83	±	8.572
744.7	CL_76:15	25.53	±	2.265	35.82	±	4.885	43.73	±	11.59	38.00	±	7.149	33.93	±	6.649	35.11	±	3.529
745.7	CL_76:14	14.36	±	2.458	26.35	±	4.744	30.05	±	4.715	19.37	±	3.881	40.76	±	10.58	34.37	±	7.619
746.7	CL_76:13	27.89	±	5.408	40.04	±	9.455	24.41	±	11.78	41.85	±	8.716	31.49	±	13.87	26.17	±	13.65
748.7	CL_76:11	23.46	±	2.730	20.48	±	6.546	23.74	±	9.140	37.48	±	9.584	8.916	±	4.266	10.01	±	3.711
750.7	CL_76:9	50.07	±	10.60	32.02	±	9.829	27.52	±	5.514	49.92	±	13.53	23.25	±	2.837	22.09	±	2.829
751.7	CL_76:8	18.46	±	2.976	15.44	±	3.887	24.06	±	6.470	22.60	±	3.894	22.94	±	3.432	21.19	±	5.182

752.7	CL_76:7	30.61	±	4.547	33.80	±	11.77	31.97	±	7.15	51.22	±	10.97	34.95	±	5.54	26.22	±	3.635
753.7	CL_76:6	14.63	±	1.469	22.30	±	3.671	13.51	±	4.312	26.33	±	6.868	13.48	±	4.012	19.81	±	4.204
754.7	CL_76:5	32.49	±	4.766	39.08	±	5.392	43.98	±	9.939	47.86	±	12.54	28.25	±	6.704	33.99	±	8.382
755.7	CL_76:4	16.71	±	3.309	22.80	±	5.542	27.35	±	7.082	27.41	±	6.046	16.80	±	4.606	20.68	±	5.676
756.7	CL_76:3	19.20	±	1.662	32.63	±	10.30	35.27	±	8.869	33.62	±	5.342	24.39	±	5.749	21.90	±	6.948
757.7	CL_76:2	12.96	±	2.558	20.29	±	2.747	20.28	±	6.098	21.11	±	3.845	29.33	±	3.867	23.75	±	3.407
758.7	CL_78:15	29.81	±	2.453	20.93	±	2.815	50.05	±	14.58	39.71	±	8.957	27.49	±	6.543	37.17	±	7.959
759.7	CL_78:14	14.67	±	2.323	19.66	±	5.099	26.42	±	7.354	22.62	±	4.630	15.70	±	2.445	14.96	±	3.549
760.7	CL_78:13	29.94	±	6.010	35.92	±	3.745	40.32	±	11.59	51.26	±	10.38	29.14	±	4.194	32.01	±	3.986
761.7	CL_78:12	14.97	±	2.549	29.11	±	8.614	21.40	±	6.417	26.83	±	4.615	28.25	±	3.080	26.00	±	5.070
762.7	CL_78:11	33.47	±	4.924	32.54	±	7.652	24.75	±	7.130	46.73	±	8.459	10.31	±	4.695	11.64	±	2.202
763.7	CL_78:10	17.58	±	3.562	20.77	±	4.255	18.73	±	5.051	22.75	±	4.868	21.84	±	5.080	15.65	±	2.272
764.7	CL_78:9	20.50	±	2.124	22.79	±	6.674	41.54	±	12.20	39.61	±	7.846	28.93	±	4.503	28.69	±	8.267
766.7	CL_78:7	27.63	±	2.025	36.36	±	8.885	33.15	±	8.752	43.28	±	8.011	22.56	±	5.010	36.97	±	5.278
767.7	CL_78:6	15.70	±	1.636	21.78	±	6.859	30.63	±	9.031	36.09	±	8.635	14.67	±	3.970	30.35	±	2.269
768.7	CL_78:5	36.33	±	6.570	28.05	±	11.41	29.72	±	10.55	36.29	±	14.17	19.05	±	3.408	13.84	±	5.176
770.7	CL_80:17	24.06	±	4.535	26.97	±	7.859	34.97	±	10.40	36.63	±	7.475	11.53	±	1.319	12.79	±	1.636
771.7	CL_80:16	15.35	±	1.054	18.32	±	3.048	17.47	±	4.826	18.50	±	4.655	27.28	±	4.623	12.31	±	3.023
772.7	CL_80:15	46.18	±	9.348	34.30	±	7.173	47.78	±	8.931	43.58	±	8.947	39.07	±	5.132	40.52	±	3.490
773.7	CL_80:14	21.07	±	2.542	38.00	±	7.310	36.47	±	7.144	27.97	±	5.209	38.04	±	5.874	39.02	±	5.223
774.7	CL_80:13	56.77	±	3.927	64.12	±	17.71	76.81	±	18.49	61.92	±	9.368	33.33	±	16.16	33.49	±	10.07
775.7	CL_80:12	34.68	±	4.693	48.83	±	14.40	62.88	±	10.31	46.60	±	6.630	25.88	±	9.105	23.49	±	4.017
776.7	CL_80:11	56.48	±	7.460	68.22	±	20.53	70.78	±	11.39	60.19	±	8.205	22.17	±	4.879	21.98	±	3.941
777.7	CL_80:10	25.58	±	2.589	42.77	±	13.07	39.19	±	16.37	28.54	±	5.716	10.56	±	4.564	18.86	±	3.100
780.7	CL_80:7	48.82	±	7.204	35.35	±	6.716	50.47	±	18.14	49.60	±	13.33	38.52	±	8.858	35.44	±	2.947
781.7	CL_80:6	23.08	±	3.830	23.33	±	4.966	38.04	±	11.39	34.00	±	10.75	27.79	±	7.665	28.35	±	4.061
782.7	CL_80:5	24.07	±	3.315	35.83	±	6.344	34.96	±	5.884	49.55	±	9.038	32.20	±	5.910	41.37	±	6.322
784.7	CL_82:17	44.35	±	12.85	22.35	±	5.028	34.97	±	4.962	41.08	±	10.38	18.34	±	7.231	23.74	±	4.376
786.7	CL_82:15	26.53	±	4.325	42.31	±	15.59	29.83	±	6.638	49.84	±	9.059	22.77	±	6.537	35.49	±	8.951
788.7	CL_82:13	25.83	±	4.079	39.79	±	8.589	35.39	±	6.900	51.77	±	12.61	44.81	±	8.121	40.81	±	5.957
789.7	CL_82:12	19.91	±	2.621	33.83	±	10.39	40.49	±	8.240	36.40	±	7.607	48.91	±	7.208	39.86	±	11.41
790.7	CL_82:11	94.44	±	21.28	72.97	±	10.76	102.1	±	21.65	72.55	±	15.30	67.13	±	10.92	53.40	±	6.846
792.7	CL_82:9	25.91	±	4.507	23.85	±	3.452	31.7	±	7.924	35.15	±	6.987	22.99	±	4.185	20.29	±	6.175
794.7	CL_84:21	37.11	±	5.591	38.81	±	4.224	44.66	±	10.55	43.83	±	7.096	31.13	±	7.690	27.30	±	6.167
795.7	CL_84:20	13.89	±	3.256	29.20	±	5.101	20.40	±	3.583	16.51	±	2.853	20.46	±	6.182	28.14	±	6.609
796.7	CL_84:19	32.33	±	7.975	34.22	±	9.388	37.56	±	10.40	40.61	±	6.456	37.79	±	6.473	30.91	±	4.790

797.7	CL_84:18	14.19	±	2.562	19.01	±	4.341	28.62	±	8.835	23.04	±	5.2	28.66	±	2.743	27.57	±	5.171
798.7	CL_84:17	23.36	±	1.638	30.71	±	5.401	38.16	±	9.429	49.32	±	12.1	29.35	±	2.638	23.82	±	5.321
799.7	CL_84:16	14.11	±	3.023	17.49	±	4.213	24.41	±	6.948	23.89	±	4.924	18.21	±	4.572	23.12	±	4.813
800.7	CL_84:15	28.76	±	3.389	21.08	±	4.957	36.18	±	8.815	42.17	±	10.16	14.34	±	2.360	26.58	±	4.742
801.7	CL_84:14	14.81	±	3.382	18.69	±	3.456	28.06	±	7.917	31.16	±	6.009	12.51	±	2.783	16.38	±	4.854
802.7	CL_84:13	29.18	±	4.994	29.77	±	9.815	37.09	±	11.81	48.00	±	10.84	22.22	±	5.177	35.50	±	7.604
803.7	CL_84:12	15.90	±	2.662	24.45	±	6.048	21.19	±	6.303	22.98	±	7.124	24.20	±	4.574	20.92	±	4.636
804.7	CL_84:11	29.40	±	2.916	40.73	±	3.576	55.37	±	12.07	47.49	±	10.01	38.71	±	5.893	36.00	±	4.756
805.7	CL_84:10	23.56	±	3.865	32.79	±	3.715	42.63	±	8.738	39.13	±	6.876	27.62	±	4.191	38.53	±	8.088
806.7	CL_84:9	44.86	±	4.534	48.57	±	14.01	68.25	±	17.15	59.87	±	9.635	47.73	±	5.969	47.35	±	6.439
808.7	CL_86:21	36.36	±	8.786	20.93	±	3.904	38.22	±	11.20	38.52	±	8.477	30.60	±	6.455	20.57	±	5.919
810.7	CL_86:19	28.66	±	3.463	36.15	±	10.24	37.04	±	7.523	45.79	±	6.476	24.03	±	6.757	30.26	±	5.044
812.7	CL_86:17	26.62	±	5.428	27.84	±	9.453	23.31	±	4.722	40.44	±	6.39	20.14	±	5.361	34.05	±	2.538
814.7	CL_86:15	27.23	±	3.864	17.63	±	5.886	30.55	±	7.034	37.01	±	6.221	30.57	±	5.498	25.68	±	4.582
815.7	CL_86:14	11.25	±	2.286	25.87	±	4.028	29.49	±	5.670	19.49	±	3.903	22.09	±	5.524	23.94	±	5.946
816.7	CL_86:13	31.39	±	3.455	31.73	±	10.57	35.96	±	10.85	39.96	±	8.458	30.18	±	3.848	21.79	±	3.358
817.7	CL_86:12	15.75	±	2.676	26.54	±	2.642	25.03	±	4.903	27.76	±	6.796	29.62	±	4.325	16.97	±	2.764
818.7	CL_86:11	27.46	±	4.686	37.18	±	7.482	44.93	±	12.41	53.86	±	11.97	33.37	±	4.254	41.18	±	5.748
819.7	CL_88:24	13.34	±	2.877	17.65	±	3.415	22.51	±	6.083	20.9	±	4.864	20.02	±	3.047	15.37	±	6.532
820.7	CL_88:23	20.14	±	3.309	28.81	±	4.263	31.79	±	6.431	39.56	±	6.728	22.66	±	8.912	27.20	±	5.140
821.7	CL_88:22	16.16	±	3.054	21.85	±	8.44	19.54	±	5.442	25.00	±	4.124	14.48	±	5.403	23.62	±	4.138
822.7	CL_88:21	21.49	±	3.056	31.86	±	7.818	33.62	±	7.899	33.32	±	6.687	34.27	±	4.951	29.83	±	7.842
824.7	CL_88:19	28.80	±	3.755	27.25	±	7.975	23.40	±	6.270	40.15	±	8.767	19.22	±	2.900	29.62	±	11.59
825.7	CL_88:18	17.18	±	4.091	15.16	±	2.634	13.15	±	3.630	20.08	±	5.631	19.17	±	5.807	28.06	±	4.962
826.7	CL_88:17	23.81	±	3.576	25.92	±	7.073	21.16	±	5.183	42.63	±	8.102	20.51	±	2.879	32.19	±	5.851
827.7	CL_88:16	9.190	±	2.134	20.83	±	6.651	19.57	±	5.339	29.16	±	6.613	16.86	±	4.557	21.55	±	2.689
832.7	CL_90:25	31.17	±	5.866	30.73	±	7.599	31.11	±	10.45	39.32	±	10.22	24.58	±	4.896	46.84	±	7.403
580.6	MLCL_52:4	9.642	±	2.424	41.32	±	6.500	21.72	±	6.665	20.28	±	4.165	24.55	±	4.086	32.84	±	4.861
581.6	MLCL_52:3	25.67	±	4.857	471.3	±	102.0	283.5	±	43.10	24.40	±	5.63	619.2	±	33.77	449.3	±	30.01
582.6	MLCL_52:2	26.49	±	4.555	294.0	±	68.28	156.7	±	13.88	29.43	±	6.799	352.9	±	29.57	223.1	±	14.65
592.6	MLCL_54:6	16.40	±	2.232	29.92	±	4.115	29.46	±	4.957	28.34	±	5.198	20.37	±	4.796	21.56	±	3.894
593.6	MLCL_54:5	25.49	±	2.793	39.32	±	6.630	44.81	±	6.467	25.98	±	3.654	46.91	±	12.29	26.90	±	5.209
594.6	MLCL_54:4	37.15	±	5.244	28.96	±	10.52	35.97	±	9.544	50.57	±	11.45	31.71	±	7.039	35.19	±	6.976
595.6	MLCL_54:3	25.60	±	2.041	202.7	±	48.11	119.0	±	24.74	22.65	±	4.261	297.3	±	17.60	220.0	±	14.07
596.6	MLCL_54:2	14.50	±	2.732	68.99	±	14.53	50.89	±	7.221	16.13	±	4.265	86.44	±	12.57	64.38	±	6.632
608.6	MLCL_56:4	17.02	±	1.178	20.05	±	3.297	29.30	±	8.220	19.39	±	4.149	18.20	±	4.242	15.45	±	3.518

616.6	MLCL_58:10	24.70	±	4.022	39.9	±	8.093	43.19	±	10.56	38.89	±	4.479	34.86	±	8.045	30.61	±	4.854
617.6	MLCL_58:9	9.223	±	1.707	19.99	±	2.188	19.72	±	3.575	15.72	±	3.387	16.88	±	2.937	9.097	±	3.367
618.6	MLCL_58:8	12.89	±	2.525	25.38	±	7.544	29.26	±	7.995	28.87	±	5.757	10.66	±	2.847	19.58	±	3.881

Supplementary Table 5: Criteria for lipid identification for each lipid class include MS mode, molecular ion, scan mode, and fragment (m/z).

**Class**	**MS Mode**	**Molecular Ion**	**Scan Mode**	**Fragment (m/z)**
CE	pos	$M+NH_4$	Precursor Ion Scan	369.3516
Cer	pos	M+H	Precursor Ion Scan	264.2686
DAG	pos	$M+NH_4$	Neutral Loss Scan	-FA
GLY	pos	M+H	Precursor Ion Scan	264.2686
LPC	pos	M+H	Precursor Ion Scan	184.0733
PC	pos	M+H	Precursor Ion Scan	184.0733
LPE	pos	M+H	Neutral Loss Scan	-141.0109
PE	pos	M+H	Neutral Loss Scan	-141.0109
TAG	pos	$M+NH_4$	Neutral Loss Scan	-FA
LPA	neg	M-H	Product Ion Scan	FA
PA	neg	M-H	Product Ion Scan	FA
LPG	neg	M-H	Product Ion Scan	FA
PG	neg	M-H	Product Ion Scan	FA
LPI	neg	M-H	Product Ion Scan	FA
PI	neg	M-H	Product Ion Scan	FA
LPS	neg	M-H	Product Ion Scan	FA
PS	neg	M-H	Product Ion Scan	FA
CL	neg	M-2H	Isotope Peak [M-2H+0.5]	

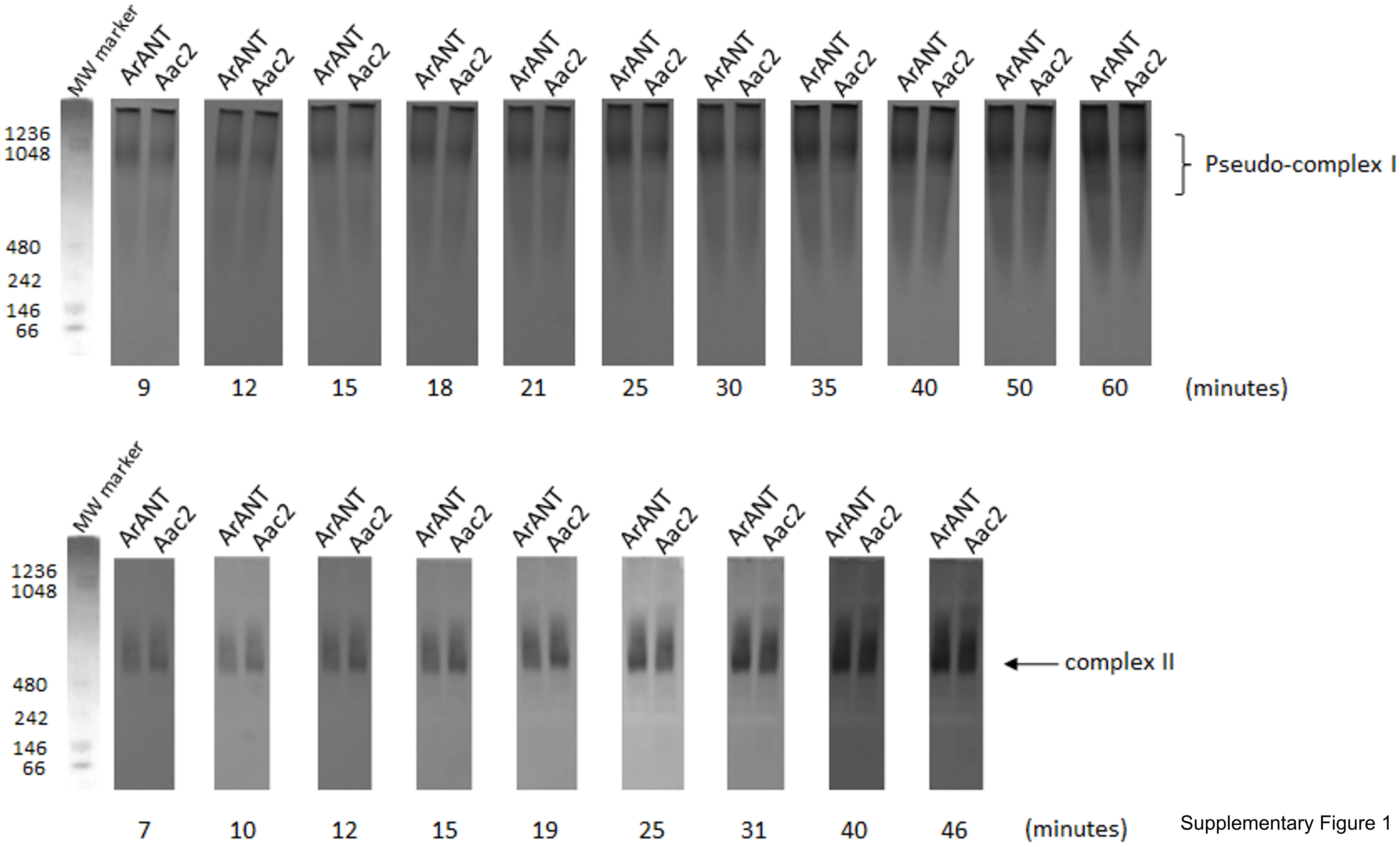

Supplementary Figure 1

Legend to Supplementary figure 1: Time-lapse of in-gel pseudo-complex I (top series) and complex II (bottom series) activities. Solubilized mitochondrial complexes (400 µg) were separated by clear-native polyacrylamide gel electrophoresis (CN-PAGE). Gels were incubated with appropriate reagents (see Materials and Methods) to visualize the activities of NADH dehydrogenases Nde1, Nde2 and Ndi1 (collectively referred to as pseudo-complex I) or succinate dehydrogenase (CII). Activity products were imaged using a standard flatbed scanner. The gel-lane scan on the top-most left corner is a molecular weight ladder (NativeMark™ Unstained Protein Standard, Thermofisher Sci, imaged at a different contrast and saturation level), to which all gel scans shown in the series can be compared to (MW marker image of both top and bottom series is the same, as these were performed sequentially). In left lane, mitochondria expressing ArANT were loaded, and in the right lane those obtained from Aac2. Incubation times are indicated in the bottom of each panel.
